# Optimized Microfluidic Synthesis of Magnesium Magnetic Silica-Based Aerogels for Pesticide Removal and Antimicrobial Water Treatment

**DOI:** 10.3390/ijms27031456

**Published:** 2026-02-01

**Authors:** Dana-Ionela Tudorache (Trifa), Alexandra-Cătălina Bîrcă, Alexandra Cristina Burdușel, Adelina-Gabriela Niculescu, Elena-Theodora Moldoveanu, Ionela C. Voinea, Miruna S. Stan, Roxana Trușcă, Bogdan Purcăreanu, Tony Hadibarata, Marius Rădulescu, Alina Maria Holban, Dan Eduard Mihaiescu, Valentin Crăciun, Alexandru Mihai Grumezescu

**Affiliations:** 1 Department of Science and Engineering of Oxide Materials and Nanomaterials, National University of Science and Technology POLITEHNICA Bucharest, 011061 Bucharest, Romaniaalexandra.birca@upb.ro (A.-C.B.); alexandra.burdusel@upb.ro (A.C.B.); adelina.niculescu@upb.ro (A.-G.N.); elena.moldoveanu99@upb.ro (E.-T.M.); truscaroxana@yahoo.com (R.T.); bogdanpb89@gmail.com (B.P.); tony.hadibarata@upb.ro (T.H.); 2 Research Institute of the University of Bucharest—ICUB, University of Bucharest, 050657 Bucharest, Romania; alina.m.holban@bio.unibuc.ro; 3 Department of Biochemistry and Molecular Biology, Faculty of Biology, University of Bucharest, 91-95 Splaiul Independentei, 050095 Bucharest, Romania; ionela-cristina.voinea@bio.unibuc.ro (I.C.V.); miruna.stan@bio.unibuc.ro (M.S.S.); 4 BIOTEHNOS SA, Gorunului Rue, No. 3-5, 075100 Otopeni, Romania; 5 Environmental Engineering Program, Faculty of Engineering and Science, Curtin University Malaysia, CDT 250, Miri 98009, Malaysia; 6 Department of Inorganic Chemistry, Physical Chemistry and Electrochemistry, National University of Science and Technology POLITEHNICA Bucharest, 1-7 Polizu Street, 011061 Bucharest, Romania; marius.radulescu@upb.ro; 7 Department of Organic Chemistry, National University of Science and Technology POLITEHNICA Bucharest, 011061 Bucharest, Romania; danedmih@gmail.com; 8 National Institute for Lasers, Plasma and Radiation Physics, 077125 Magurele, Romania; valentin.craciun@inflpr.ro

**Keywords:** magnetic aerogels, silica aerogels, vortex-microfluidic, microfluidic synthesis, water decontamination, pesticide adsorption, antimicrobial potential

## Abstract

Water represents the fundamental source of life for all human and animal populations; however, its consumption has become increasingly hazardous due to high levels of pollution. Modern agricultural practices rely heavily on pesticides, which significantly contribute to water contamination and imbalances in aquatic ecosystems. Moreover, another critical category of pollutants consists of pathogenic bacteria that proliferate in aquatic environments, mainly originating from hospital and urban wastewater because of human activity. Considering these major environmental and health challenges, the present study aims to develop an optimized method for water treatment by synthesizing magnetic silica-based aerogels using a microfluidic vortex chip and systematically varying synthesis parameters to enhance material performance. The physicochemical properties of the aerogels were characterized using XRD, FTIR, SEM, EDS, and BET. The pesticide adsorption capacity of the materials was evaluated using FT-ICR HR-MS analysis, which demonstrated the high efficiency of the aerogels in removing a complex mixture of pesticides. In parallel, antimicrobial efficacy was assessed against *E. faecalis*, *E. coli*, and *P. aeruginosa* isolated from surface water, hospital wastewater, and the influent of a well-known wastewater treatment plant in Bucharest, as well as against ATCC reference strains. Additionally, the study investigated the biocompatibility and biological responses of magnetic aerogels using MTT assays, nitric oxide production, lactate dehydrogenase release, intracellular ROS levels, and quantification of total protein, malondialdehyde, and reduced glutathione in HaCaT and HEK293 cell lines. The results confirm the efficiency and application potential of the developed materials and emphasize the importance of optimizing synthesis to achieve high-performance aerogels for effective decontamination of polluted waters.

## 1. Introduction

Globally, water pollution represents one of the most pressing environmental challenges, with major implications for human health and aquatic ecosystems. Water pollution can generally be classified into two main categories: point-source pollution and nonpoint-source pollution. Point-source pollution refers to contamination originating from a clearly identifiable, localized source, such as effluents discharged from industrial facilities, factory drainage pipes, oil spills from tankers, or wastewater released from sewage treatment plants. These sources contribute to wastewater that flows through storm sewers and eventually contaminates large aquatic areas. In contrast, non-point-source pollution arises from multiple, diffuse, and often unidentifiable sources, making it difficult to control. Such pollution is commonly associated with the use of agricultural pesticides, residues from industrial fertilizers, and other harmful by-products of agricultural and domestic activities. These contaminants are gradually dispersed into the soil, leading primarily to groundwater pollution. The major sources of water pollution include industrial and agrochemical waste, nutrient enrichment (eutrophication), radioactive waste, sewage and other oxygen-demanding materials, acid rain, sediment disruption, climate change, oil spills, invasive species, thermal pollution, and accelerated urbanization [1,2,3,4].

One of the most significant and persistent sources of water pollution is agricultural activity, present in both developed and developing regions. As the global population continues to grow, the demand for food production increases, accordingly, leading to the intensification of agricultural practices and, consequently, to a substantial rise in the use of chemical fertilizers, pesticides, and other environmentally hazardous substances. In agriculture, herbicides, fungicides, and insecticides are commonly used for preventive or curative treatments with broad-spectrum activity. However, the widespread use of these pesticides inevitably contaminates groundwater, river systems, marine environments, lakes, and oceans. These pollutants readily enter aquatic systems, primarily through surface run-off and tile drainage, processes that are significantly intensified by heavy precipitation events. Such contamination causes adverse effects on both aquatic life and the sources of drinking water for terrestrial organisms. The intensity of pesticide transport is strongly influenced by climatic, geological, and hydrological factors that govern the mobility and dispersion of these compounds in the environment [5,6]. A study conducted by Fiona H. M. Tang et al. [7] highlighted the global risk of pesticide pollution, revealing that approximately 74.8% of the world’s agricultural land is subject to some degree of pesticide contamination.

Pesticides can be classified by chemical composition into several major groups, each exhibiting distinct properties that influence their environmental behavior and biological effects. The most commonly used classes include organochlorine, organophosphate, carbamate, and pyrethroid compounds, known for their high efficacy but varying toxicity and persistence. Other categories comprise biologically or botanically derived substances, inorganic copper-based pesticides, and specialized groups such as triazines, urea derivatives, and dithiocarbamates [8,9,10].

These chemical substances, found in the food and water consumed by living organisms, exert severe adverse effects on biological systems. Pesticides generate reactive oxygen species, and the levels of antioxidants and the natural cellular defense mechanisms against oxidative stress are consequently reduced. This imbalance can lead to inflammation and epigenetic dysregulation. Long-term exposure to pesticides and the resulting oxidative imbalance may contribute to the development of neurodegenerative diseases, carcinogenesis, and various respiratory, cardiovascular, renal, reproductive, and endocrine disorders. In addition, acute exposure to pesticides can cause immediate health effects, including skin and eye irritation, dizziness, nausea, and headaches [11,12,13]. At the same time, numerous infectious diseases such as gastroenteritis, diarrhea, cholera, and dysentery occur as a consequence of consuming water contaminated with pathogenic microorganisms, often leading to fatal outcomes. Water contamination with such pathogens is primarily anthropogenic, resulting from the presence of fecal matter containing multiple microorganisms. Once introduced into sewage systems through wastewater overflows during heavy rainfall or uncontrolled discharges, these pathogens can reach water sources and pollute them. Unlike chemical pollution caused by pesticides or other toxic substances, which leads to chronic and cumulative effects, biological contamination results in acute illnesses [14,15,16,17].

Regarding the presence of fecal bacteria, commonly referred to as fecal coliforms, they can be classified into three main categories: commensal, extraintestinal, and diarrheagenic, including genera such as Klebsiella, Citrobacter, Hafnia, Enterobacter, and Escherichia. Among these, the most prevalent and clinically significant is *Escherichia coli* (*E. coli*). Although *E. coli* naturally inhabits the gastrointestinal tracts of humans and animals, it can act as a biological pollutant in the environment, being transmitted through fecal matter, particularly in hospital settings involving infected individuals [18,19,20]. Like *E. coli*, another bacterium naturally found in the gastrointestinal tract, *Enterococcus faecalis* can adversely affect water quality through fecal contamination. This microorganism is recognized as a causative agent of urinary tract infections, burn and surgical wound infections, bacteremia, endocarditis, and abdominal and biliary tract infections. Moreover, *E. faecalis* is frequently detected on catheters and other implanted medical devices, acting as an important opportunistic pathogen. However, it is among the most commonly identified and abundant bacterial species in hospital wastewater [21,22,23,24]. Another notable bacterium is *Pseudomonas aeruginosa*, a nosocomial pathogen commonly detected in wastewater from healthcare facilities. It can persist in water, soil, and plants, and is responsible for numerous hospital-acquired infections, especially in patients with cystic fibrosis, as well as for respiratory, urinary tract, wound, and soft tissue infections, and infections affecting patients with thermal injuries. Transmission may occur through contact with contaminated water (e.g., during showering or bathing), through medical instruments (e.g., surgical equipment or endoscopes), and through non-sterile aqueous antiseptic solutions. A particularly concerning feature of *P. aeruginosa* is its high resistance to common antibiotics and its ability to form biofilms, which enhance its persistence and make eradication from hospital and environmental settings extremely challenging [25,26,27,28].

Given the growing concerns about water pollution and its direct impact on public health, the present study builds on our previous research, aiming to develop innovative materials capable of mitigating or even resolving these critical issues. In our previous work, we established the feasibility of on-chip synthesis of salicylic-acid-functionalized magnetite nanoparticles (Fe_3_O_4_–SA) and their subsequent incorporation into silica aerogels, yielding advanced adsorbents with high relevance for pesticide removal from water and with encouraging initial biocompatibility profiles. Three-dimensional microfluidic platforms (vortex/spiral) enabled precise control over Fe_3_O_4_–SA size and dispersity and the fabrication of magnetically responsive silica aerogels with uniform morphology and high extraction efficiencies for representative pesticides, as confirmed by comprehensive physicochemical analyses and by in vitro cytocompatibility assessments of on-chip-produced materials [29,30,31].

Given the reported ability of divalent cations such as Mg^2+^ to promote network crosslinking and enhance pollutant binding in hybrid silica materials [32,33,34,35,36], the present study incorporates magnesium ions into the Fe_3_O_4_–SA/silica aerogel system to explore their contribution to pesticide adsorption and antimicrobial performance. In this context, the current study focuses on optimizing the synthesis method for magnesium magnetic aerogels using a vortex-type microfluidic system to assess the efficiency of the functionalized materials for water decontamination. The demonstration of efficacy includes the evaluation of pesticide adsorption capacity via FT-ICR mass spectrometry, the assessment of antimicrobial activity against microorganisms isolated from hospital, urban, and surface wastewater, as well as the biocompatibility evaluation using HEK293 and HaCaT cell lines.

## 2. Results

This section presents the results obtained from the characterization of the 11 magnesium magnetic aerogel samples (Sample 1–Sample 11), synthesized microfluidically by varying the concentrations of the reactant solutions. The purpose of this analysis is to provide an integrated comparative overview of the materials and to identify the differences arising from the modified synthesis parameters.

**Figure 1:**
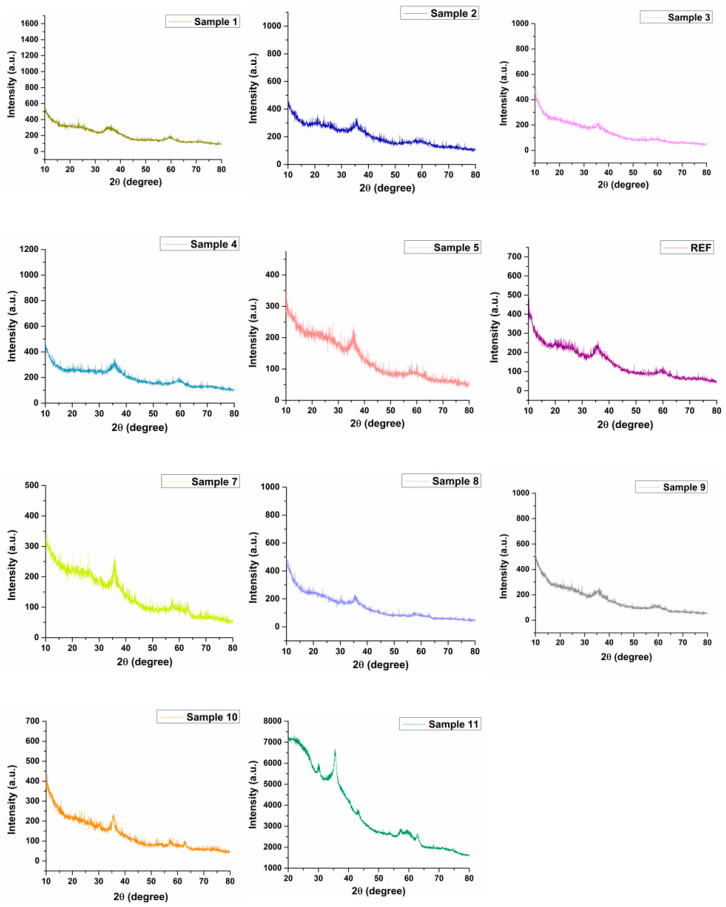
XRD diffractograms obtained for all 11 magnetic aerogel samples (Sample 1–Sample 11).

Figure 1 presents the XRD diffractograms obtained for the 11 magnesium magnetic aerogel samples. Although subtle variations can be observed among the samples, the overall crystallographic pattern remains largely similar, as expected, given that the same types of reactant solutions were used during the synthesis of the magnesium magnetic aerogels. With the exception of Sample 1, which exhibits a diffraction pattern characteristic of a poorly crystalline material due to its composition (see Table 1), all other samples confirm the presence of magnetic nanoparticles through the main diffraction maximum located around 35° (2θ), consistent with the PDF-ICDD reference card 04-012-7038 for Fe_3_O_4_, corresponding to the (311) Miller index. Among them, Samples 5, 7, 10, and 11 display diffraction maxima at 36.093°, 35.308°, 35.276°, and 35.272° (2θ), respectively, with comparable intensities and the sharpest peak profiles, indicating higher crystallinity than the other samples. This observation correlates with the synthesis parameters: Sample 5 was produced using 75% of the maximum Fe_3_O_4_-SA concentration (60 mg/mL), whereas Sample 7 represents the first numerically guided experimental optimization, containing 47 mg/mL Fe_3_O_4_-SA. Sample 10 was synthesized using the highest concentration of Fe_3_O_4_–SA nanoparticles (70 mg/mL), whereas Sample 11 displays a value very close to that of Sample 7 with respect to their magnetic nanoparticle content, being formulated at 47 mg/mL. A notable difference between these samples is the presence of reflections near 57° and 62° (2θ), characteristic of magnetite and corresponding to the (511) and (440) Miller indices, which are observed in Samples 7 and 11. This distinction can be attributed to the variation in alginate content: Sample 5 contains 262 mg/mL ALG, whereas Sample 7 contains only 50 mg/mL, suggesting a higher degree of crystallinity in Sample 7. Moreover, Sample 10 was synthesized using an ALG concentration of 250 mg/mL, whereas Sample 11 was prepared with an ALG concentration of 160 mg/mL. The presence of SiO_2_ in all samples is confirmed by the broad halo observed within 25–27° (2θ), in agreement with PDF-ICDD reference cards 01-073-3460 and 01-077-8622. In samples containing higher Mg^2+^ concentrations, additional weak peaks appear within the 2θ range of 43–48°, consistent with the presence of MgO. These peaks are of low intensity and partially overlap with the characteristic reflections of Fe_3_O_4_, making them difficult to distinguish visually. Nonetheless, their positions match the reference patterns for MgO (PDF-ICDD 04-006-0699 and PDF-ICDD 01-078-4523). Sample 11 exhibits the most distinct XRD pattern among all formulations, likely due to the combined effect of its moderate Fe_3_O_4_–SA loading (50 mg/mL), intermediate alginate concentration (160 mg/mL), and the relatively high Mg^2+^ content (2300 mg/mL) used during synthesis. This specific composition appears to promote enhanced structural organization within the silica network.

**Figure 2:**
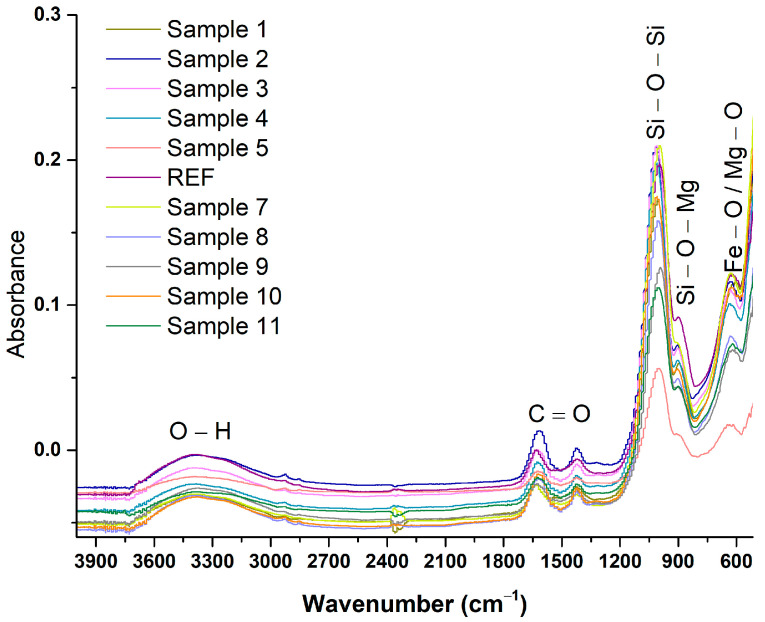
FTIR spectra obtained for all 11 magnetic aerogel samples (Sample 1–Sample 11).

Figure 2 shows the FTIR spectra recorded for the 11 magnesium magnetic aerogel samples synthesized in this study. All samples exhibit similar functional groups, corresponding to the reactants used in the synthesis. The broad band in the 3600–3000 cm^−1^ region, assigned to O–H stretching vibrations, arises from adsorbed water and hydroxyl groups within the material. The absorption band at 1744 cm^−1^ corresponds to the C=O stretching vibration, while the signal at 1487 cm^−1^ is associated with the aromatic C=C skeletal vibrations of salicylic acid [37]. Given the silica-based aerogel structure, the intense band near ~1100 cm^−1^ is attributed to the asymmetric stretching of Si–O–Si linkages [38,39,40]. Around ~900 cm^−1^, Si–O stretching vibrations are observed, but contributions from Si–O–Mg bonds may also be present due to interactions between Mg^2+^ ions and the silica network [41,42]. In the low-wavenumber region, between 550 and 600 cm^−1^, the characteristic metal–oxygen (M–O) stretching vibrations are detected, reflecting the presence of Fe- and Mg-based oxide phases [43,44,45,46,47,48,49,50]. Overall, the FTIR findings are consistent with and complementary to the XRD analysis, confirming the structural features of the synthesized aerogels.

**Figure 3:**
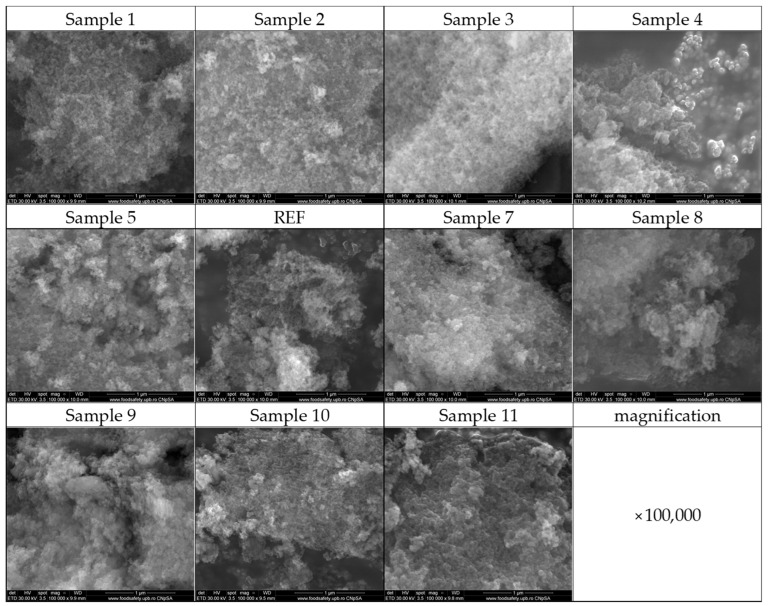
SEM micrographs acquired at 100,000× magnification for all 11 magnesium magnetic aerogel samples (Sample 1–Sample 11).

Next, the magnesium magnetic aerogel samples were examined by SEM to assess morphology. The micrographs shown in Figure 3 confirm the successful formation of the characteristic silica aerogel morphology, consisting of fine nanoscale particles interconnected into a three-dimensional porous network. The typical silica aerogel texture is evident in all samples, although subtle differences in particle aggregation and surface features can be observed.

**Figure 4:**
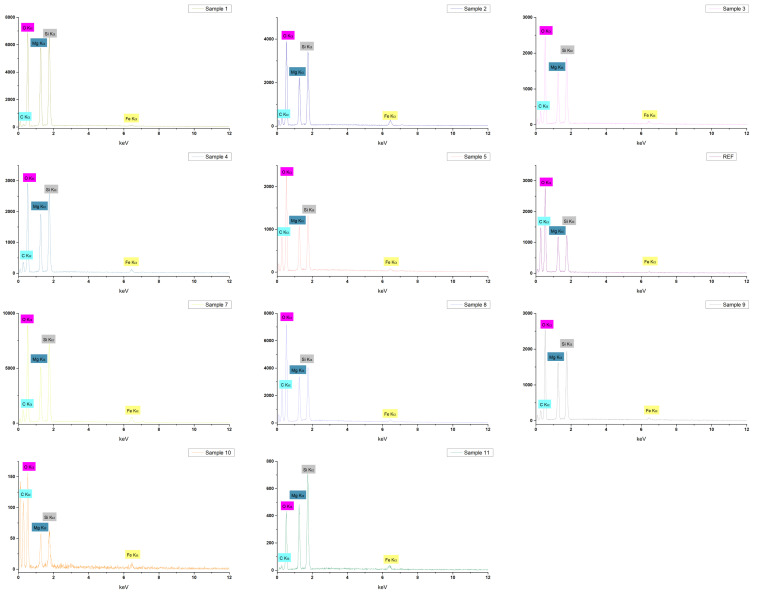
EDS results obtained for all 11 magnesium magnetic aerogel samples (Sample 1–Sample 11).

The elemental composition of the samples was assessed using EDS coupled to the scanning electron microscope, and Figure 4 displays the EDS spectra for all 11 magnetic aerogel samples. Consistent with the formulation of the materials obtained by varying the concentrations of the reactant solutions, each sample exhibits characteristic signals for carbon, oxygen, magnesium, silicon, and iron. This elemental distribution confirms the incorporation of all intended components into the final structure of the magnesium magnetic aerogels.

**Figure 5:**
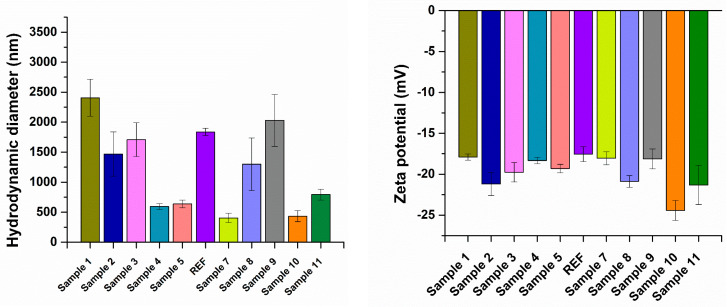
DLS result expressed as hydrodynamic diameter (nm) and zeta potential (mV) obtained for Sample 1–Sample 11.

Subsequently, the aerogel formulations were analyzed by DLS to evaluate their behavior in liquid media. The DLS results provide information on the hydrodynamic characteristics of the aerogels in suspension. With respect to the hydrodynamic diameter (Figure 5), the samples in the theoretically designed series (Sample 1–REF) generally exhibited larger values than those in the numerically guided, experimentally optimized series (Sample 7–Sample 11) (Table 1). Zeta potential measurements revealed negative surface charge for all aerogel formulations, attributed to the anionic nature of the silica matrix and alginate components. The zeta potential values ranged from −17.6 mV to −24.4 mV (Table 2), indicating moderate colloidal stability of the aerogel dispersions, with a generally favorable tendency toward electrostatic stabilization in aqueous media.

**Figure 6:**
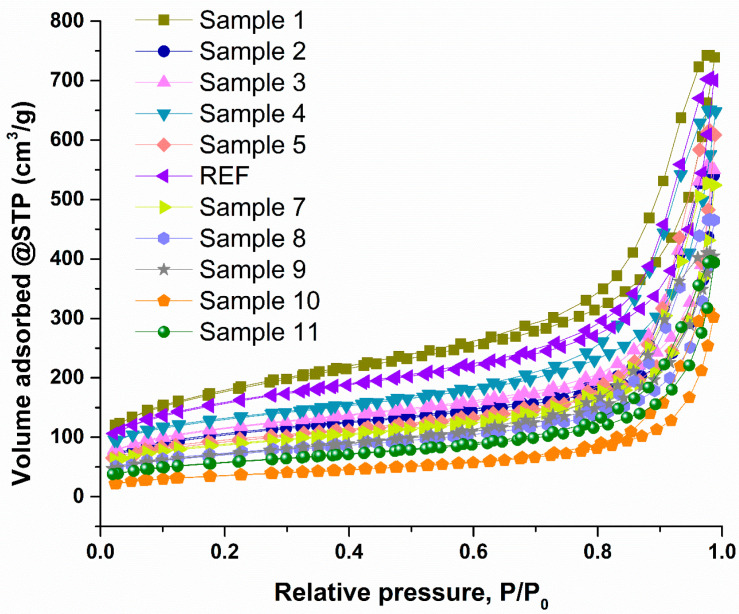
N2 adsorption/desorption isotherm for all 11 magnesium magnetic aerogel samples (Sample 1–Sample 11).

Given the nature of the materials developed and optimized in this study, BET analysis is essential for evaluating N_2_ adsorption isotherms, the pore structure of the samples, and the quantitative parameters of specific surface area, pore volume, and pore diameter. Figure 6 shows the N_2_ adsorption isotherms for all samples, which fall into the type IV category according to the International Union of Pure and Applied Chemistry (IUPAC) classification. This type of isotherm, characterized by an “S-shaped” adsorption–desorption branch and a pronounced increase at higher relative pressures, is typical of mesoporous materials. The specific surface areas of the samples exhibit variability, ranging from 127.8 m^2^/g for Sample 10 to 615.7 m^2^/g for Sample 1. The highest surface areas are observed for Sample 1 (615.7 m^2^/g), REF (539.5 m^2^/g), Sample 4 (436.3 m^2^/g), Sample 2 (357.1 m^2^/g), Sample 3 (391.8 m^2^/g), and Sample 5 (315.2 m^2^/g). Conversely, the lowest surface areas are recorded for Sample 10 (127.8 m^2^/g), Sample 11 (200.2 m^2^/g), Sample 8 (243.7 m^2^/g), Sample 7 (294.3 m^2^/g), and Sample 9 (250.0 m^2^/g). These results indicate that the samples generated during the first optimization stage (Sample 1–Sample 5), based on controlled proportional variations in reactant concentrations (minimum, maximum, 25%, 50%, and 75%), exhibited superior properties. In contrast, the samples obtained from the numerically guided optimized formulations (Sample 7–Sample 11) displayed lower specific surface areas. The pore volume follows the same trend as the surface area: samples from Sample 1 to REF exhibit a fine structure with a well-developed porous network, whereas Samples 7–11 show reduced pore volumes, suggesting a denser and more compact framework. Nevertheless, all 11 samples maintain pore diameters within the mesoporous range, according to IUPAC standards, ranging from 6.10 nm (Sample 1) to 10.28 nm (Sample 10). This confirms the formation of a fine silica-based network and highlights the suitability of these materials for adsorption applications, particularly for the removal of pesticides from water.

A clearer insight into the influence of reactant mass concentrations on the textural properties, namely specific surface area, pore volume, and average pore diameter, can be derived from the data presented in Table 3. The results indicate that simultaneous increases in all reactant concentrations decrease the specific surface area, accompanied by reductions in pore volume and increases in average pore diameter. For Samples 7–11, generated through numerically guided experimental optimization, a more compact aerogel structure is suggested. This behavior contrasts with that observed for Samples 1–REF, in which a highly aerated, well-developed porous network is preserved.

To demonstrate the applicability of the magnesium magnetic aerogels for water decontamination through pesticide adsorption, their performance was evaluated using FT-ICR HR-MS analysis. Table 4 summarizes the key mass spectrometric parameters required for the identification of each pesticide, including the *m*/*z* values, ion form, and charge state (z). These data confirm the presence of each compound in the initial pesticide mixture and enable the quantification of residual concentrations following treatment with the 11 magnesium magnetic aerogel formulations.

Table 5 presents the extraction efficiency values, expressed as percentages, calculated from the FT-ICR HR-MS results. When evaluating each aerogel formulation individually for each pesticide, it becomes evident that several samples exhibit consistently high adsorption performance. These include Sample 5, Sample 7, Sample 8, and Sample 11, all of which were synthesized using relatively high concentrations of Mg^2+^ ions (approximately 2000 mg/mL), except for Sample 7, where the Mg^2+^ concentration was 1810 mg/mL but the ALG content was at its minimum. Conversely, some samples demonstrated fluctuating performance, showing good efficiency with certain pesticides but low removal capacity with others. This behavior was observed in Samples 1, 3, and 4. Notably, Sample 1 and Sample 4 correspond to the minimum and 25% concentration levels within the predefined variation interval, while Sample 3 contains a high concentration of ALG, with Mg^2+^ and Fe_3_O_4_-SA concentration levels set at 50% of their respective ranges. Furthermore, the reference sample (REF), synthesized according to the original formulation, exhibits markedly reduced extraction efficiencies for most pesticides, underscoring the need to optimize the aerogel composition to enhance adsorption performance.

**Figure 7:**
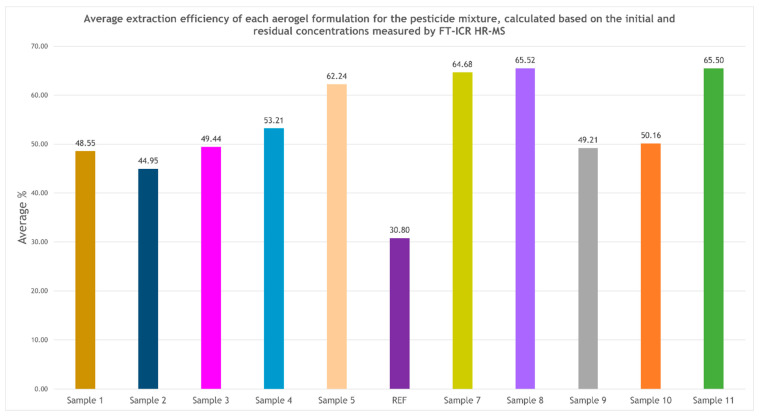
Graphical representation of average extraction efficiency of each aerogel formulation for the pesticide mixture, calculated based on the initial and residual concentrations measured by FT-ICR HR-MS.

The graphical representation of the average extraction efficiency values (Figure 7), calculated as the mean removal efficiency for all pesticides tested in each sample, highlights four aerogel formulations with superior performance, each exceeding 60% average efficiency. These formulations, Sample 8 (65.52%), Sample 11 (65.50%), Sample 7 (64.68%), and Sample 5 (62.24%), emerge as the most promising candidates for water decontamination applications. The other magnesium magnetic aerogels also exhibit stable adsorption capacities, though slightly lower than those of the top-performing samples. Overall, the optimization process led to substantial improvements, with several formulations achieving more than double the extraction efficiency of the original reference sample (REF).

The next step in demonstrating the applicability of the synthesized aerogels is to evaluate their antimicrobial effectiveness. The bacterial species included in this study were strategically selected to represent a range of ecologically and clinically relevant contexts, enabling a comparative assessment of the antimicrobial activity of the aerogel formulations under investigation. Accordingly, strains belonging to *E. faecalis* (Gram-positive), *E. coli*, and *P. aeruginosa* (both Gram-negative) were analyzed, originating from natural, anthropogenic, and clinical environments, alongside standardized reference strains from international culture collections.

For the determination of the minimum inhibitory concentration (MIC), the analysis was conducted separately for each bacterial species, and the results were grouped by MIC for *E. faecalis*, *E. coli*, and *P. aeruginosa*.

**Figure 8:**
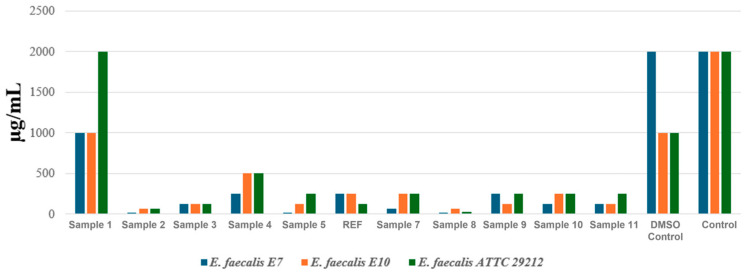
Minimum inhibitory concentration values for the Sample 1–Sample 11 magnesium magnetic aerogel samples tested against three *E. faecalis* strains: E7 and E10 (environmental isolates) and ATCC 29212 (reference strain). Results are expressed in μg/mL and represent the lowest concentration at which complete bacterial growth inhibition was observed after 24 h of incubation. DMSO and untreated bacterial controls are included for comparison. Bacterial inoculum was standardized to 0.5 McFarland.

The first set of results, shown in Figure 8, corresponds to the evaluation performed on three *Enterococcus faecalis* strains, each originating from a different source. Two strains (E7 and E10) were isolated in 2024 from Danube River water, representing naturally occurring isolates, while the third strain is an international reference strain, ATCC 29212.

The results demonstrated that several aerogel samples exhibited strong antibacterial activity against the *E. faecalis* strains tested. The most pronounced effects were observed for Sample 2, Sample 5, and Sample 8. For instance, in the case of *E. faecalis* strain 1 (E7, isolated from the Danube), the MIC value dropped substantially to 15.625 μg/mL for these formulations, indicating a remarkable ability to inhibit bacterial growth. This is particularly significant, as the controls (DMSO and untreated strain) showed MIC values of 2000 μg/mL, confirming the absence of inhibitory effects in the absence of the active material. The second natural isolate, *E. faecalis* 2 (E10), exhibited a similar sensitivity profile. Once again, Sample 2 and Sample 8 were the most effective, with MIC values of 62.5 μg/mL and 125 μg/mL, respectively, demonstrating clear antibacterial activity, although slightly lower than for strain E7. The reference strain *E. faecalis* 3 (ATCC 29212) showed a sensitivity pattern comparable to that of the environmental isolates. Sample 2 and Sample 8 produced the strongest inhibitory effects, with MIC values of 62.5 μg/mL and 31.25 μg/mL, respectively, confirming that the antimicrobial efficacy of these formulations is not strain dependent. Additionally, other formulations, such as Sample 3 and Sample 11, displayed moderate activity, with MIC values ranging from 125 to 250 μg/mL.

In the next experiment, four *E. coli* strains were evaluated, each originating from distinct sources. Three strains (CCE1D, DCE2D, and CE1D) corresponding to *E. coli* 1, 2, and 3 were isolated in July 2024 from water samples collected downstream of the Glina wastewater treatment plant, while the fourth strain (*E. coli* 4—ATCC 25922) represents an internationally recognized reference strain widely used in antimicrobial susceptibility testing.

**Figure 9:**
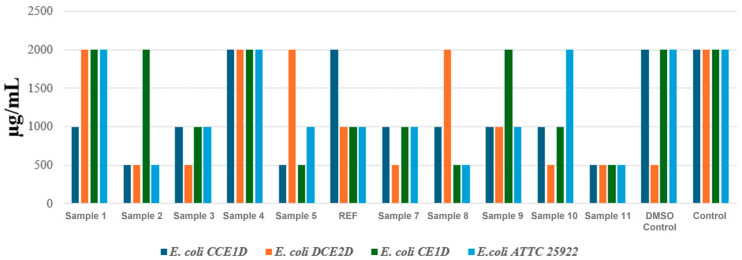
Minimum inhibitory concentration values for the Sample 1–Sample 11 magnesium magnetic aerogel samples tested against four *E. coli* strains: CCE1S, DCE2D, and CE1D (environmental isolates) and ATCC 25922 (reference strain). Results are expressed in μg/mL and represent the lowest concentration at which complete bacterial growth inhibition was observed after 24 h of incubation. DMSO and untreated bacterial controls are included for comparison. Bacterial inoculum was standardized to 0.5 McFarland.

The results shown in Figure 9 revealed moderate variability in the susceptibility of *E. coli* strains to the aerogel formulations. MIC values ranged from 500 to 2000 μg/mL, indicating lower antibacterial activity than observed against *E. faecalis*. Both the DMSO control and the untreated strain displayed MIC values of 2000 μg/mL, confirming the absence of inhibitory effects in the absence of the active material. Among the tested formulations, Samples 2, 5, and 11 demonstrated the most consistent antimicrobial activity, particularly against the environmental isolates. For instance, in the case of *E. coli* 1 (CCE1D), these formulations reduced the MIC to 500 μg/mL, indicating a notable inhibitory effect. *E. coli* 2 (DCE2D) exhibited a similar response, with the same formulations achieving MIC values of 500 μg/mL. *E. coli* 3 (CE1D) showed a higher degree of resistance, with MIC values between 1000 and 2000 μg/mL for most aerogels. However, it responded favorably to Sample 5 and Sample 8, which yielded MIC values as low as 500 μg/mL. For the reference strain ATCC 25922 (*E. coli* 4), sensitivity was comparable to that of the environmental isolates, particularly for Samples 2, 8, and 11, which produced MIC values of 500–1000 μg/mL.

In the final MIC assessment experiment, three *P. aeruginosa* strains were tested, each originating from ecologically and clinically distinct sources: a strain isolated from untreated urban wastewater collected from the influent of the Glina treatment plant (*P. aeruginosa* 1), a strain isolated from hospital effluents collected at the Sf. Paraschiva Infectious Diseases Hospital in Iași (*P. aeruginosa* 2), and the international reference strain ATCC 27853 (*P. aeruginosa* 3). The objective was to determine whether the aerogel formulations could exert antimicrobial activity against a species well known for its intrinsic multidrug resistance.

**Figure 10:**
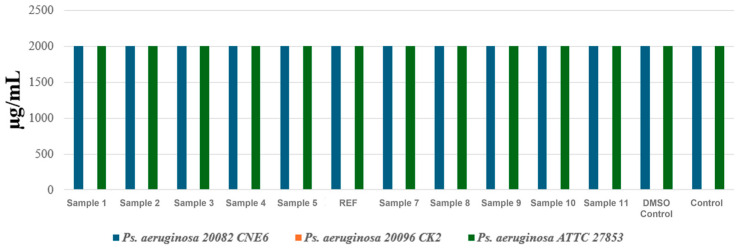
Minimum inhibitory concentration values for the Sample 1–Sample 11 magnesium magnetic aerogel samples tested against three *P. aeruginosa* strains: 20082 CNE6 and 20096 CK2 (environmental isolates) and ATCC 27853 (reference strain). Results are expressed in μg/mL and represent the lowest concentration at which complete bacterial growth inhibition was observed after 24 h of incubation. DMSO and untreated bacterial controls are included for comparison. Bacterial inoculum was standardized to 0.5 McFarland.

The results represented in Figure 10 showed that all aerogel formulations were ineffective against the tested *P. aeruginosa* strains. Both the urban isolate and the ATCC reference strain displayed constant MICs of 2000 μg/mL across all samples, including the negative controls, indicating a complete lack of inhibitory activity. Moreover, for the hospital-derived strain, no MIC value could be determined within the tested range, as no antimicrobial response was detected, reflecting an extremely high resistance phenotype. This observation aligns with the existing literature, as *P. aeruginosa* possesses highly efficient membrane barriers, robust biofilm-forming ability, and active efflux systems that prevent intracellular accumulation of antimicrobial agents [51,52,53,54].

Compared with the results obtained for *E. faecalis* and *E. coli*, in which several aerogel formulations demonstrated clear inhibitory effects, *P. aeruginosa* proved substantially more resistant. Even the ATCC reference strain showed no measurable susceptibility, confirming that the aerogels tested in their current form do not exhibit antibacterial activity against this species.

**Figure 11:**
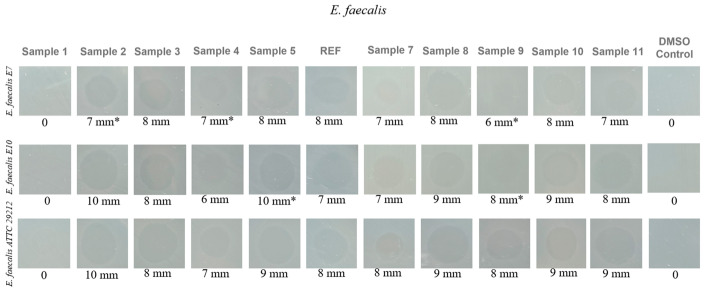
Inhibition zone diameter (mm) for the Sample 1–Sample 11 magnesium magnetic aerogel samples tested against three *E. faecalis* strains: E7 and E10 (environmental isolates) and ATCC 29212 (reference strain). Values represent the mean inhibition halo measured after 18–24 h of incubation at 37 °C. Negative controls (DMSO and untreated bacterial control) are included for comparison. * indicate samples where bacterial colonies were visible inside the growth inhibition zone.

The inhibition zone diameter assay highlights the antibacterial performance of the three *E*. *faecalis* strains (E7, E10, and ATCC 29212), as shown in Figure 11. The quantitative assessment reveals clear differences in the antimicrobial activity of the aerogel formulations, which are directly influenced by the reactant concentrations used during synthesis. As anticipated, Sample 1 exhibited no inhibition zones for any of the strains, confirming its lack of antimicrobial activity. In contrast, Samples 2, 5, 8, 10, and 11 produced substantial inhibition zones ranging from 9 to 10 mm for at least two of the tested strains. Among these, Samples 2 and 5 demonstrated the strongest activity, generating 10 mm inhibition zones against strains E10 and ATCC 29212. These findings are fully consistent with the previously determined MIC values. Samples 3, 4, 7, and 9 also displayed antimicrobial effects, although to a lesser extent, producing inhibition zones of 7–8 mm.

**Figure 12:**
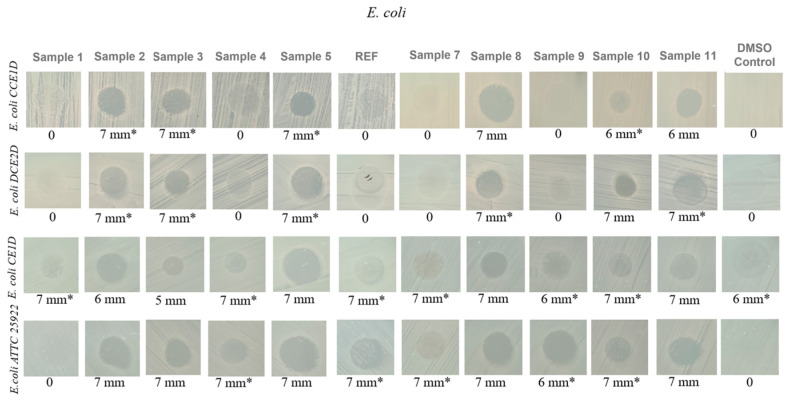
Inhibition zone diameter (mm) for the Sample 1–Sample 11 magnesium magnetic aerogel samples tested against four *E. coli* strains: CCE1S, DCE2D, and CE1D (environmental isolates) and ATCC 25922 (reference strain). Values represent the mean inhibition halo measured after 18–24 h of incubation at 37 °C. Negative controls (DMSO and untreated bacterial control) are included for comparison. * indicate samples where bacterial colonies were visible inside the growth inhibition zone.

For the inhibition zone results obtained for the four *E. coli* strains (Figure 12), the data indicate a genuine yet limited antibacterial effect. Samples 2, 5, 8, 10, and 11 produced inhibition zones of approximately 7 mm for several of the tested *E. coli* strains, suggesting a minimal inhibitory effect, essentially a borderline response.

**Figure 13:**
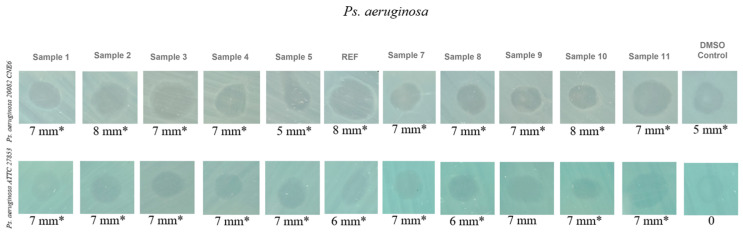
Inhibition zone diameter (mm) for the Sample 1–Sample 11 magnesium magnetic aerogel samples tested against two *P. aeruginosa* strains: 20082 CNE6 (environmental isolates) and ATCC 27853 (reference strain). Values represent the mean inhibition halo measured after 18–24 h of incubation at 37 °C. Negative controls (DMSO and untreated bacterial control) are included for comparison. * indicate samples where bacterial colonies were visible inside the growth inhibition zone.

The inhibition zone assessment for *P. aeruginosa*, including both the environmental isolate and the reference strain (Figure 13), indicates a lack of true antibacterial activity, consistent with the MIC results. The small 6–8 mm zones observed in some samples are comparable to those of the control and are most likely due to physical diffusion or wetting effects rather than genuine bacterial growth inhibition.

**Figure 14:**
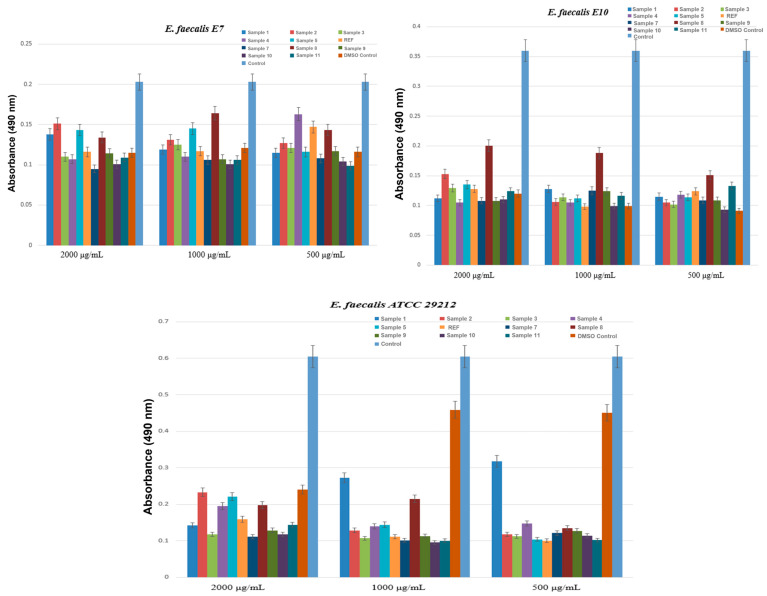
Biofilm formation inhibition by the Sample 1–Sample 11 magnesium magnetic aerogel samples tested against three *E. faecalis* strains: E7 and E10 (environmental isolates) and ATCC 29212 (reference strain). Absorbance values at 490 nm reflect the biofilm biomass formed after 24 h of incubation in the presence of the aerogels at the three tested concentrations (2000, 1000, and 500 μg/mL). Significant absorbance reductions compared to the bacterial control indicate effective anti-biofilm activity. Data are presented as mean ± SD (*n* = 3).

The results of the biofilm-formation inhibition assay for the tested aerogels are presented below. The data are shown graphically and grouped by bacterial strain: *E. faecalis*, *E. coli*, and *P. aeruginosa*. The results obtained for biofilm inhibition in the three *E. faecalis* strains (E7, E10, and ATCC 29212), shown in Figure 14, highlight the anti-biofilm activity of the aerogel formulations. In all three cases, the absorbance values recorded for the treated groups were significantly lower than those for the untreated strain control, indicating a substantial and genuine reduction in biofilm biomass. Similar activity patterns were observed for all three strains, with the aerogel formulations reducing biofilm formation at all three tested concentrations (2000, 1000, and 500 μg/mL). Overall, the most effective formulations for inhibiting *E. faecalis* biofilm were Samples 1, 2, 3, 4, 7, 9, 10, and 11, which consistently inhibited biofilm across all tested concentrations. These results indicate that the anti-biofilm effect of the aerogels is not limited to a particular source of bacterial isolation or to the reference strain.

**Figure 15:**
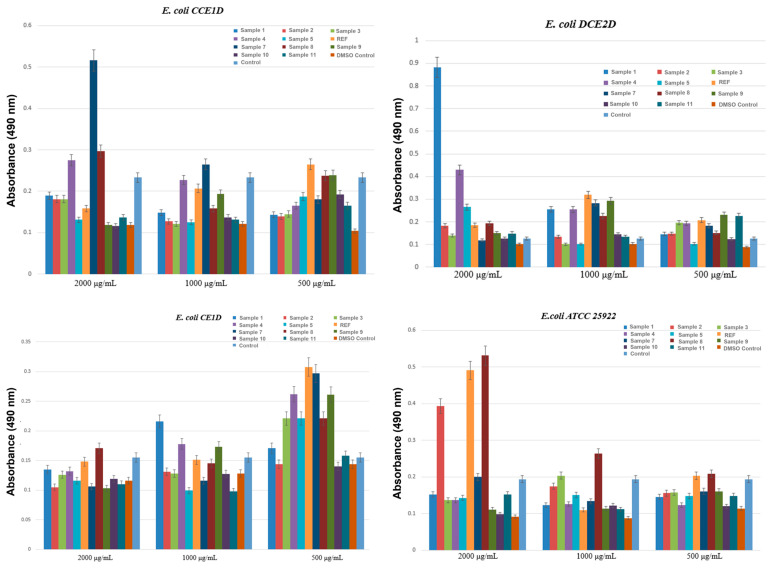
Biofilm formation inhibition by the Sample 1–Sample 11 magnesium magnetic aerogel samples tested against four *E. coli* strains: CCE1S, DCE2D, and CE1D (environmental isolates) and ATCC 25922 (reference strain). Absorbance values at 490 nm reflect the biofilm biomass formed after 24 h of incubation in the presence of the aerogels at the three tested concentrations (2000, 1000, and 500 μg/mL). Significant absorbance reductions compared to the bacterial control indicate effective anti-biofilm activity. Data are presented as mean ± SD (*n* = 3).

Figure 15 presents the results of biofilm inhibition by *E. coli* across the three environmental isolates and the standard strain. In this case, clear differences are observed compared with the effects recorded for *E. faecalis*. For the *E. coli* CCE1D strain, the results indicate effective biofilm inhibition starting from the lowest concentration tested, 500 µg/mL. However, the effects shift with increasing concentration: at 500 µg/mL, Samples 1–5, 7, 10, and 11 show biofilm inhibition; at 1000 µg/mL, Samples 4 and 7 tend to become less effective; and at 2000 µg/mL, Samples 4 and 7 display absorbance values higher than the strain control, while Samples 9, 10, and 11 produce a marked decrease in biofilm biomass, consistent with antimicrobial activity. Thus, for this strain, the strongest anti-biofilm effects at 2000 µg/mL are observed for Samples 9, 10, and 11. For the *E. coli* DCE2D strain, the results support the antimicrobial properties of the aerogels at 2000 µg/mL for Sample 7, at 1000 µg/mL for Samples 3 and 5, and at 500 µg/mL for Sample 5. In the *E. coli* CE1D strain, a general increase in absorbance is observed as concentration decreases, indicating weaker biofilm inhibition at lower concentrations. At 2000 µg/mL, most samples exhibit absorbance values comparable to or lower than those of the strain control, demonstrating antibiofilm activity. At 1000 µg/mL, the differences become more pronounced, with Samples 5, 7, and 11 maintaining lower absorbance values, suggesting persistent inhibitory activity. At 500 µg/mL, the concentration is insufficient to demonstrate clear anti-biofilm efficacy in the aerogels. For the standard strain *E. coli* ATCC 25922, at 2000 µg/mL, most aerogels show no meaningful antimicrobial effect. However, as the concentration decreases, improved responses are observed: at 1000 µg/mL, lower absorbance values are recorded for all samples except Samples 8, 3, and 2; whereas at 500 µg/mL, all samples except Samples 8 and 6 demonstrate inhibitory effects on biofilm formation.

**Figure 16:**
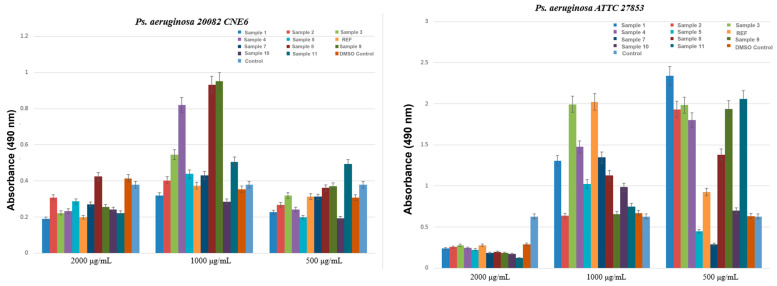
Biofilm formation inhibition by the Sample 1–Sample 11 magnesium magnetic aerogel samples tested against two *P. aeruginosa* strains: 20082 CNE6 (environmental isolates) and ATCC 27853 (reference strain). Absorbance values at 490 nm reflect the biofilm biomass formed after 24 h of incubation in the presence of the aerogels at the three tested concentrations (2000, 1000, and 500 μg/mL). Significant absorbance reductions compared to the bacterial control indicate effective anti-biofilm activity. Data are presented as mean ± SD (*n* = 3).

The samples tested against *P. aeruginosa* strains showed concentration-dependent inhibition of biofilm formation. As shown in Figure 16, for the environmental isolate *P. aeruginosa* 20082 CNE6 at 2000 µg/mL, all samples, except Sample 8, showed lower absorbance than the bacterial control, indicating a significant reduction in biofilm biomass. As the concentration decreased, a clear decline in antimicrobial performance was observed. At 1000 µg/mL, only Sample 10 showed absorbance values lower than the control, suggesting partial inhibitory activity. At 500 µg/mL, most samples exhibited absorbance values comparable to or slightly lower than the control, with the strongest reduction in biofilm formation recorded for Samples 5 and 10. For the reference strain *P. aeruginosa* ATCC 27853, an anti-biofilm effect was evident only at the highest concentration (2000 µg/mL), where the absorbance at 490 nm was markedly reduced relative to the bacterial control. At lower concentrations, no clear inhibitory effect was observed, except for Samples 5 and 7 at 500 µg/mL, which showed slightly lower absorbance values than the control.

Given the biodiversity present in water sources requiring treatment with the materials developed in this study, and the overarching goal of improving water quality for safe domestic use, assessing the interactions between the magnesium magnetic silica aerogels and mammalian cells is critical. Accordingly, the following section presents the results of the biocompatibility evaluation of all 11 aerogel formulations using two representative cell lines, HaCaT and HEK293, selected to determine their cytocompatibility and potential effects on cell viability.

**Figure 17:**
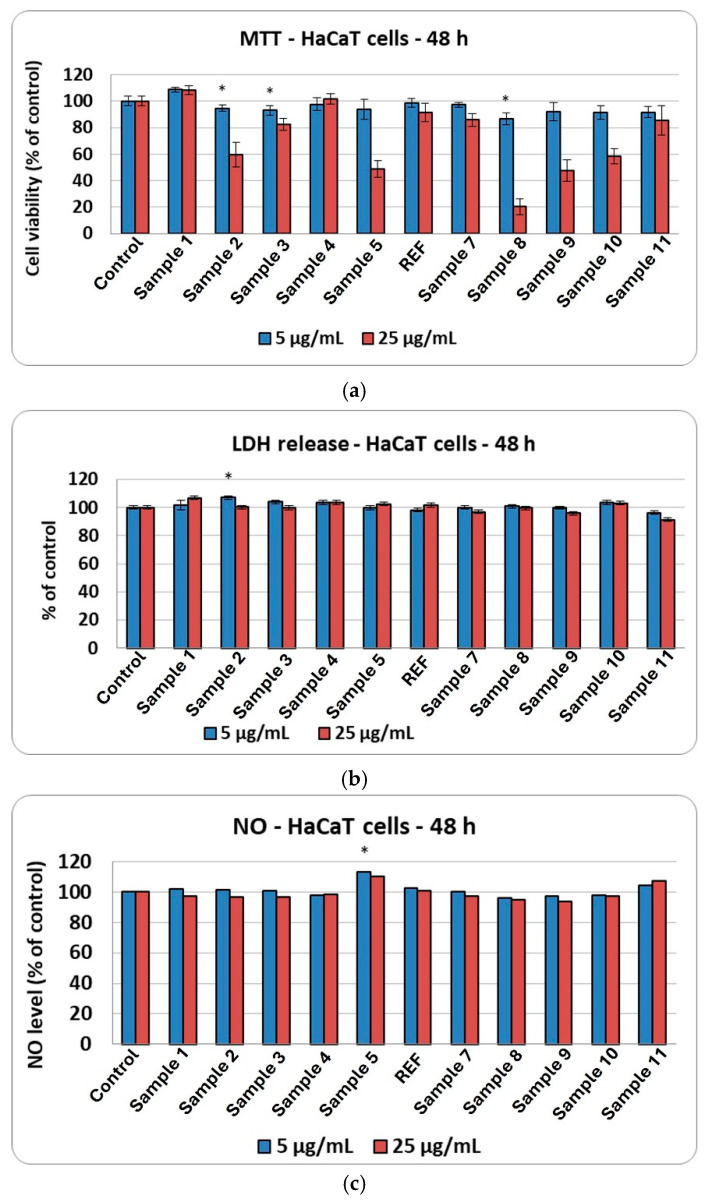
Graphical representations of (**a**) HaCaT cell viability, (**b**) LDH release, and (**c**) NO levels after 48 h of incubation with Sample 1–Sample 11 magnesium magnetic silica aerogels. Results are expressed as the mean of three replicates ± standard deviation and presented relative to the control. * *p* < 0.05 compared to the control.

Figure 17a shows the effects of the 11 aerogel formulations on HaCaT cells’ viability after 48 h of exposure. The MTT assay results highlight clear differences depending on the concentration tested. Sample 1 showed slightly higher cell viability at both 5 and 25 µg/mL, exceeding that of the untreated control. Overall, at 5 µg/mL, most aerogel formulations have maintained cell viability values close to those of the control, with only minor variations between samples. However, increasing the concentration to 25 µg/mL resulted in a pronounced decrease in viability for several formulations. The lowest viability percentages were observed in Samples 5, 8, and 9, indicating a dose-dependent cytotoxic effect. Conversely, some formulations, including Sample 4, REF, Sample 7, and Sample 11, preserved relatively higher cell viability at 25 µg/mL, although still below the control level. These results suggest that certain aerogel compositions are better tolerated by HaCaT cells, while others induce a substantial reduction in viability at higher concentrations.

Figure 17b presents the results of the LDH release assay performed on cells exposed to the 11 aerogel samples. There were no significant changes compared to the control, regardless of the aerogel formulation or the concentration tested, indicating the absence of membrane-damaging cytotoxic effects.

**Figure 18:**
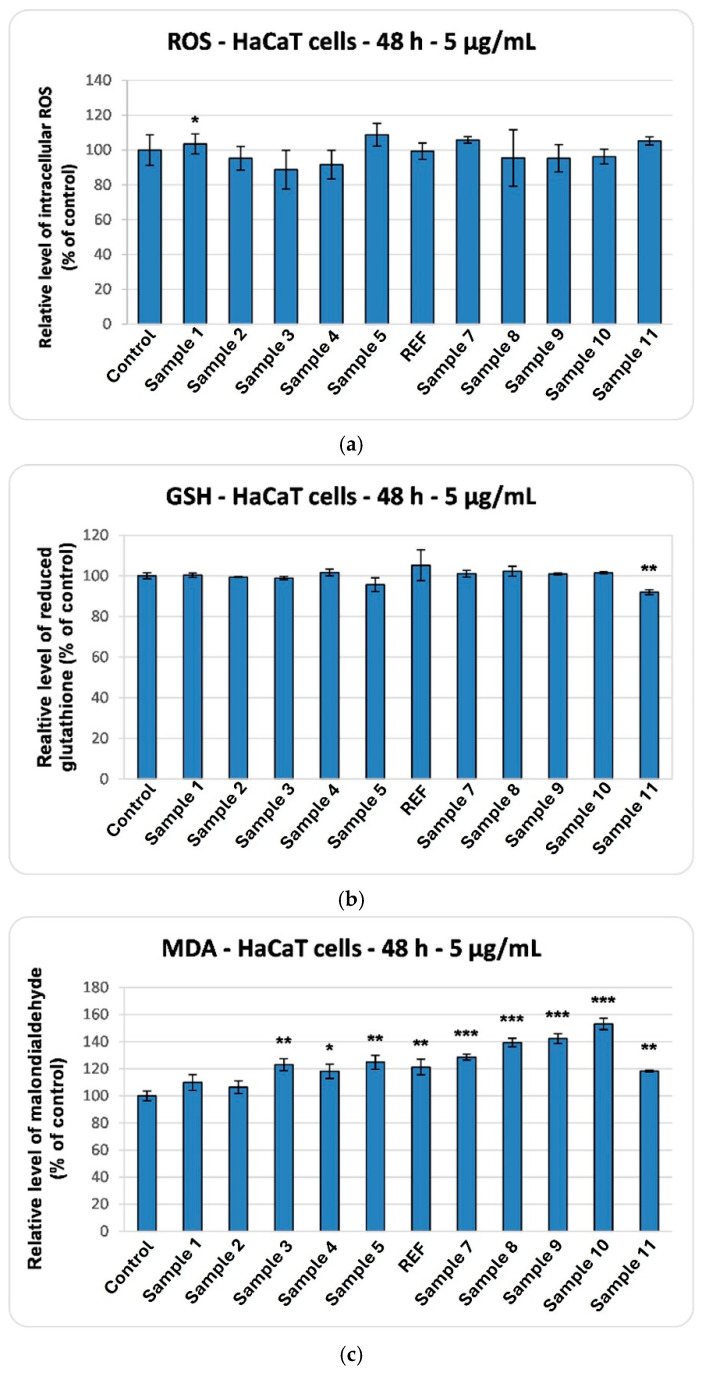
Graphical representations of (**a**) ROS production, (**b**) GSH, and (**c**) MDA levels in HaCaT cells after 48 h of incubation with 5 µg/mL of Sample 1–Sample 11 magnesium magnetic silica aerogels. Results are expressed as the mean of three replicates ± standard deviation and presented relative to the control. * *p* < 0.05, ** *p* < 0.01, and *** *p* < 0.001 compared to the control.

Figure 17c shows the nitric oxide production in cells treated with the 11 aerogel samples. The levels remained close to those of the control, indicating the absence of any detectable inflammatory response. This positive profile of the synthesized aerogels is further supported by the ROS production results shown in Figure 18a. The recorded values are close to the control ones, although the best outcomes were observed for Sample 3, while Sample 5 exhibited the least favorable response.

The biocompatibility of the samples was further supported by the assessment of cellular GSH levels, which, as shown in Figure 18b, exhibited no significant changes compared to the control. Conversely, the presence of aerogels (Sample 10) in the culture medium induced an increase in MDA levels of up to 50% above control, as presented in Figure 18c. Even in the absence of major alterations in overall ROS or GSH levels, certain aerogel formulations may trigger localized lipid peroxidation through direct physicochemical interactions with the cell membrane, as reflected by elevated MDA levels, without substantially disrupting the cell’s global redox balance.

**Figure 19:**
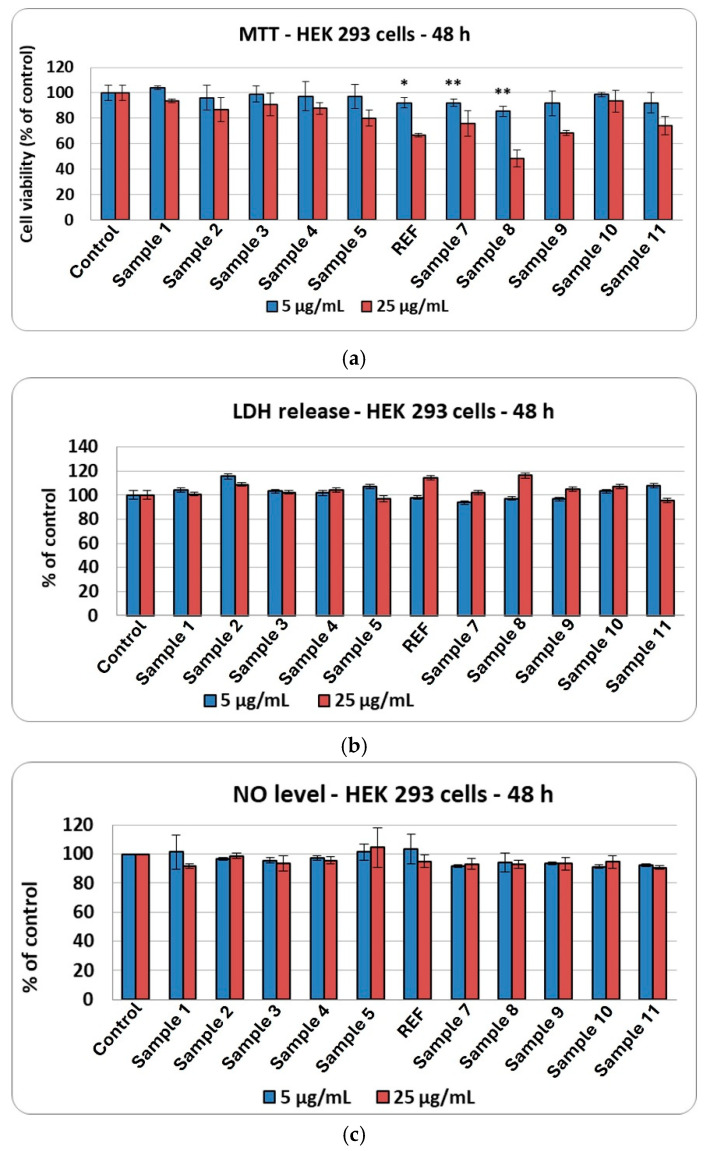
Graphical representations of (**a**) HEK cell viability, (**b**) LDH release, and (**c**) NO levels after 48 h of incubation with Sample 1–Sample 11 magnesium magnetic silica aerogels. Results are expressed as the mean of three replicates ± standard deviation and presented relative to the control. * *p* < 0.05 and ** *p* < 0.01 compared to the control.

Subsequently, the same set of assays assessing the biological response to the synthesized aerogels was performed on the HEK293 cell line. Cell viability after incubation with the aerogels remained comparable to the control across all samples at 5 µg/mL. In contrast, exposure to 25 µg/mL led to a reduction in viable cells only for samples 8 and 9 (Figure 19a). Consistent with the results obtained for HaCaT cells, no increase in LDH (Figure 19b) or nitric oxide (Figure 19c) release was detected relative to the control, demonstrating the absence of membrane damage and the lack of an induced inflammatory response.

**Figure 20:**
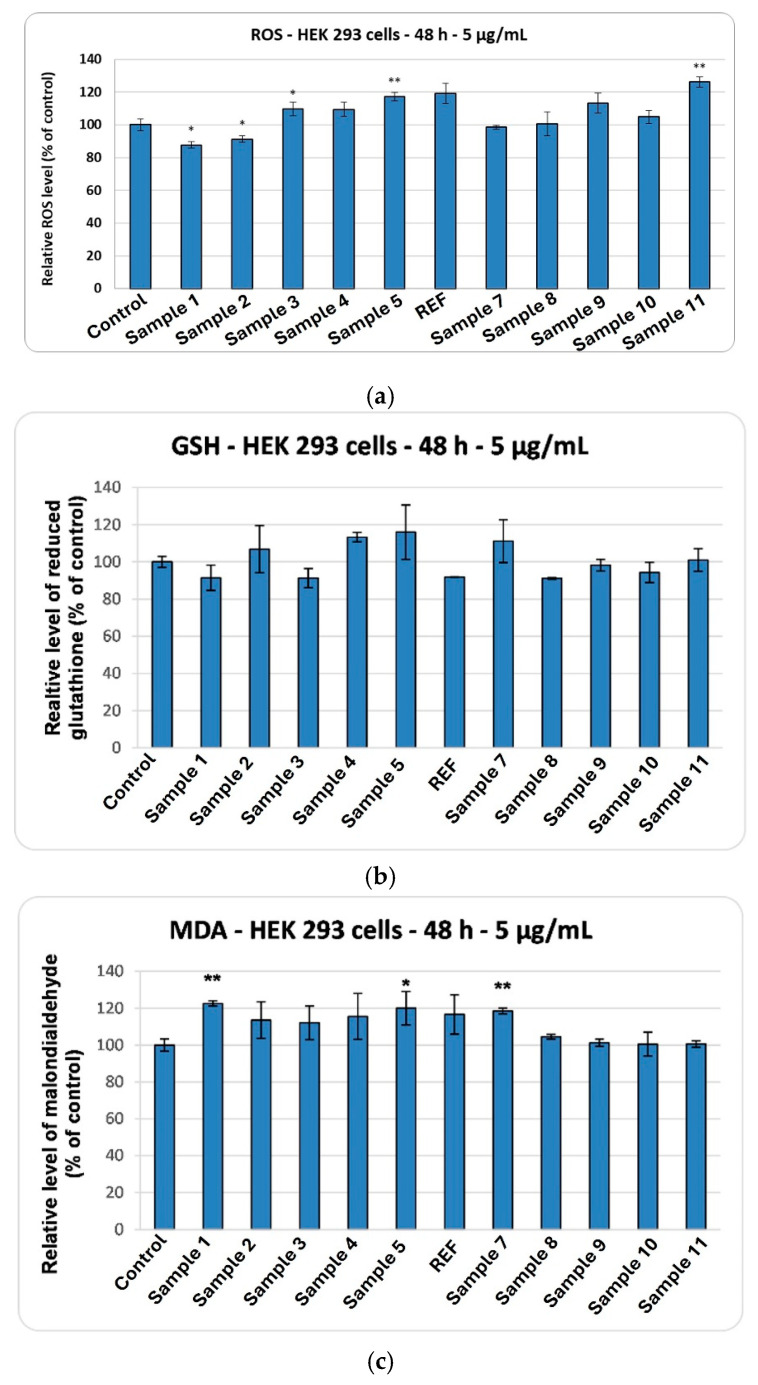
Graphical representations of (**a**) ROS production, (**b**) GSH, and (**c**) MDA levels in HEK cells after 48 h of incubation with 5 µg/mL of Sample 1–Sample 11 magnesium magnetic silica aerogels. Results are expressed as the mean of three replicates ± standard deviation and presented relative to the control. * *p* < 0.05 and ** *p* < 0.01 compared to the control.

Concerning ROS production (Figure 20a), the values remained close to those of the control for Samples 1, 2, 7, and 8, whereas slight increases were observed following incubation with Samples 5 and 11. Furthermore, after 48 h of exposure to the magnesium magnetic silica aerogels, HEK 293 cells showed no significant change in GSH levels (Figure 20b) compared to the control, indicating no major alterations in overall cellular redox balance. However, MDA levels increase by up to 120% relative to control in several aerogels (Sample 1–5, REF, Sample 7), indicating a subtle activation of lipid peroxidation (Figure 20c), much lower than that observed in HaCaT cells. However, this dissociation between redox markers and lipid damage suggests a possible physicochemical interaction of the aerogels with the cell membrane, selectively affecting membrane lipids without inducing a detectable systemic oxidative response. Overall, the results indicate localized oxidative stress, primarily affecting membrane integrity rather than eliciting a global oxidative response.

**Figure 21:**
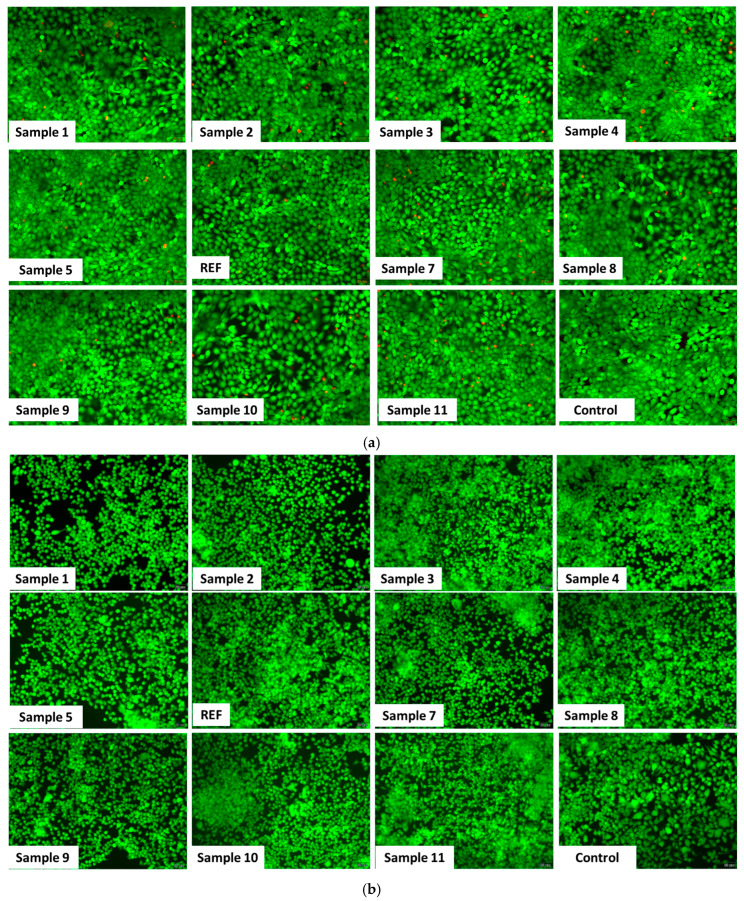
Representative fluorescence microscopy images showing live cells (stained with Calcein-AM, green) and dead cells (stained with propidium iodide, red) using the Live/Dead assay after 48 h of incubation of (**a**) HaCaT or (**b**) HEK293 cell lines with aerogels (5 µg/mL). Scale bar: 50 µm.

Figure 21 provides qualitative insights into cell viability from fluorescence microscopy images of HaCaT cells (Figure 21a) and HEK293 cells (Figure 21b) stained with the Live/Dead assay. Following incubation with 5 µg/mL of the aerogels, a higher number of dead cells (stained red with propidium iodide) can be observed in human keratinocytes compared with renal cells. Nevertheless, the proportion of viable cells (stained green with Calcein AM) remains comparable to that in the control, and these observations are consistent with the results of the MTT assay.

**Figure 22:**
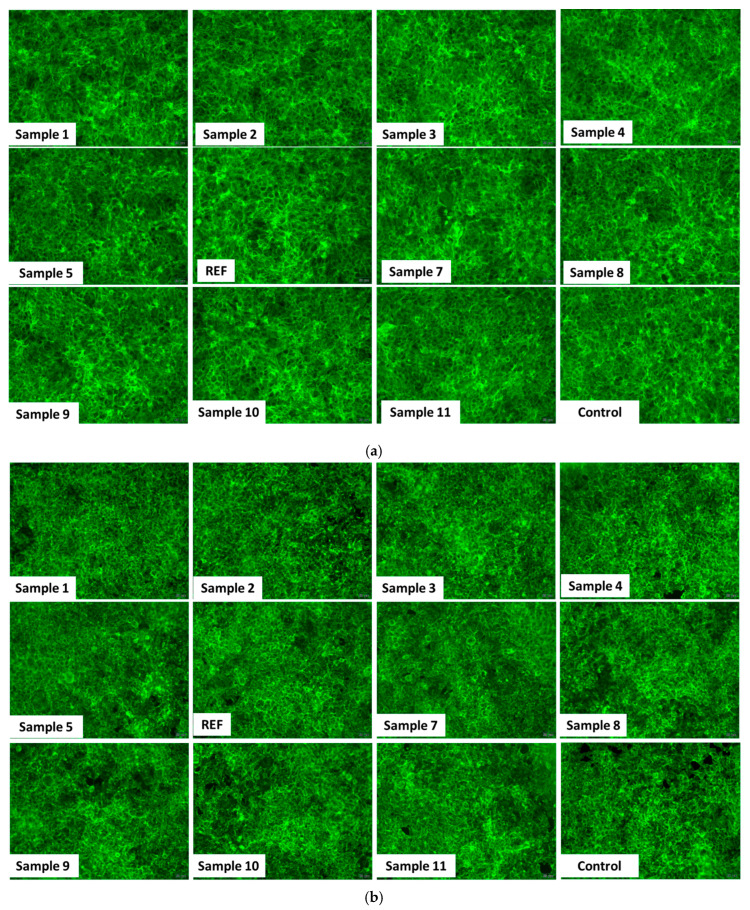
Representative fluorescence microscopy images showing the actin cytoskeleton (stained with phalloidin-FITC, green) after 48 h of incubation of (**a**) HaCaT or (**b**) HEK293 cell lines with aerogels (5 µg/mL). Scale bar: 50 µm.

Figure 22 illustrates the organization of the actin cytoskeleton (green) in HaCaT cells (Figure 22a) and HEK293 cells (Figure 22b) following exposure to 5 µg/mL of the aerogels. In both cell lines, actin filaments display a normal distribution, with well-defined adhesion structures and overall cellular organization comparable to those of the untreated controls. These representative images indicate that the aerogels do not impair cellular mechanics or structural integrity, further supporting their biocompatibility at this tested concentration.

## 3. Discussion

As there is a growing need for innovative materials capable of simultaneously addressing both chemical and microbiological water contamination, the present study focuses on the optimization of a multifunctional material that efficiently adsorbs pesticides while inhibiting bacterial growth. The material is a silica-based aerogel enhanced with ALG, Mg^2+^ ions, and Fe_3_O_4_-SA nanoparticles, each component contributing distinctly to the improved performance of the final system.

The XRD patterns revealed distinct structural variations among the magnesium magnetic silica aerogel samples as a function of reactant concentration. The aerogel composition comprises both a silica matrix and ALG, and the diffraction features associated with Fe_3_O_4_-SA and Mg^2+^-containing phases are modulated by these constituents [55]. It is well established that increasing the silica or polymer content can restrict crystal growth, leading to peak broadening and an apparent reduction in crystallinity, an effect further amplified by the particles’ nanometric size [56]. The identification of magnesium-related phases is additionally challenging due to partial overlap with magnetite diffraction peaks, a phenomenon commonly reported in hybrid oxide systems [57,58,59]. Among the eleven aerogel samples investigated, Sample 11 exhibits the most distinct diffraction pattern, suggesting an optimal balance of reactant concentrations during synthesis. This composition likely promotes improved crystallite development and/or more effective integration within the silica network. In this context, the observed variations in diffraction peak characteristics are both expected and meaningful, reflecting the outcome of the optimization process. Selecting an appropriate compositional balance is therefore crucial for developing multifunctional aerogels with tailored structural features and enhanced functional performance, particularly for advanced water decontamination applications.

Similar principles to those discussed for the XRD analysis also apply to the FTIR results, as compositional variations directly influence the materials’ spectral signatures. It is well established that changes in ALG concentration, which impart hydrophilicity to the material and are crucial for the adsorption of polar pesticides, lead to increased absorbance intensity of the broad band around ~3200 cm^−1^, associated with O–H stretching vibrations [60,61]. The formation of the silica network was confirmed by the characteristic absorption band in the 1080–1100 cm^−1^ region, corresponding to Si–O–Si vibrations. Furthermore, the identification of an additional band attributed to Si–O–Mg linkages suggests that Mg^2+^ ions are incorporated into the silica framework rather than forming separate magnesium oxide phases. These findings are consistent with the literature on magnesium-doped silica systems and on the formation of metal–oxygen–silicon bridges [62,63,64]. Overall, the FTIR results corroborate the successful integration of silica, alginate, Mg^2+^ ions, and Fe_3_O_4_–SA nanoparticles within a unified hybrid structure. The observed functional groups and bonding environments support the multifunctional behavior of the aerogels and help explain their enhanced adsorption and antimicrobial performance.

The optimization process, achieved by varying the mass concentrations of the reactant solutions, is also reflected in the morphostructural features of the aerogels, as revealed by SEM micrographs. A major distinction can be observed between the Sample 1–REF series, whose compositions were defined theoretically, and the Sample 7–Sample 11 series, whose reactant concentrations were determined by numerically guided experimental optimization. The Sample 1–REF aerogels exhibit a highly aerated, well-defined silica network, characterized by fine, interconnected structures typical of mesoporous aerogels. In contrast, Samples 7–11 exhibit more compact morphologies, with pronounced agglomerations that limit the formation of an open, aerated surface. These observations are directly correlated with the sample optimization process, as variations in the mass concentrations of the reactant solutions significantly influence the resulting aerogel morphology [65,66,67]. The DLS analysis further supports these observations, as the hydrodynamic diameter values obtained for the theoretically designed series (Sample 1–REF) are consistently higher than those measured for the numerically guided experimentally optimized series (Sample 7–Sample 11). This behavior suggests that, in aqueous media, the Sample 1–REF formulations tend to form larger, interconnected structures or aggregates, whereas the Sample 7–Sample 11 aerogels are more likely to undergo partial structural fragmentation, resulting in smaller and more dispersed agglomerates. In addition, the influence of synthesis parameters is also reflected in the zeta potential measurements. Several samples from the numerically guided experimentally optimized series exhibited absolute zeta potential values exceeding 20 mV, indicating enhanced electrostatic stabilization in suspension. Notably, a clear correlation with Fe_3_O_4_-SA and Mg^2+^ content can be identified, as all zeta potential values above 20 mV were recorded for Sample 2 (−21.20 mV), Sample 8 (−20.89 mV), Sample 10 (−24.44 mV), and Sample 11 (−21.30 mV). These samples correspond to formulations containing the highest combined concentrations of Mg^2+^ ions and Fe_3_O_4_–SA nanoparticles (see Table 6 and Table 7). This trend suggests a synergistic contribution of Mg^2+^ ions and Fe_3_O_4_–SA nanoparticles to the surface charge density and colloidal stability of the aerogel dispersions.

The BET analysis results are consistent with the general properties of silica-based aerogels, which typically exhibit N_2_ adsorption isotherms of type IV, characteristic of mesoporous materials. In addition, key textural parameters such as specific surface area, pore volume, and average pore size are strongly influenced by the aerogel synthesis conditions. Variations in precursor concentrations induce noticeable changes in the BET results characteristics, specifically, increasing the overall precursor concentration leads to a decrease in both specific surface area and porosity. This behavior can be attributed to faster reaction kinetics at higher concentrations, which promote network densification and may result in more fragile or compact structures [68,69,70]. In aerogels incorporating ALG within their structure, the effect of the polymer on textural properties can be dual. On the one hand, ALG may increase the overall surface area by promoting network expansion and hydrophilicity; on the other hand, polymer addition generally enhances the mechanical stability of the material. However, the final outcome strongly depends on the nature of the polymer and the synthesis environment, as ALG behavior can vary significantly across solvent systems and crosslinking conditions [56,68,71,72]. CTAB is well recognized for its role as a structure-directing agent that promotes porosity in silica-based materials. Consequently, variations in CTAB concentration also influenced the BET results obtained in this study. In general, higher CTAB concentrations favor the formation of interconnected porous networks, thereby increasing the specific surface area [68,73]. Nevertheless, the present results indicate that porosity development is not solely governed by CTAB concentration. For instance, the highest specific surface area (615.7 m^2^/g) and pore volume (0.939 cm^3^/g) were obtained for Sample 1, where no CTAB was used. In this case, the minimal presence of nanoparticles allowed the silica network to form without additional structural interference, resulting in a highly aerated structure. The reference sample (REF) exhibited the second-highest surface area (539.5 m^2^/g) and pore volume (0.845 cm^3^/g), containing 800 mg/mL CTAB. Notably, the same CTAB concentration was employed for Sample 10, which displayed a substantially lower surface area (127.8 m^2^/g) and pore volume (0.329 cm^3^/g). This comparison clearly demonstrates that multiple factors, such as the combined effects of polymer content, nanoparticle loading, ionic species, and overall solid concentration, must be considered when designing and optimizing aerogel materials, as they collectively govern the final properties.

Regarding the FT-ICR HR-MS results, a clear trend emerges: pesticides with bulky aromatic structures, such as pyrazophos, triazophos, and propyzamide, tend to be adsorbed more efficiently than smaller halogenated compounds, which exhibit more variable adsorption responses [74,75]. This behavior is consistent with stronger π–π interactions, increased hydrophobic affinity, and enhanced coordination with surface-active sites present within the aerogel matrix [76,77]. Moreover, aerogel formulations synthesized at concentrations determined during the numerically guided experimental optimization stage consistently outperformed those synthesized at theoretically selected concentrations. These findings highlight that the reference aerogel did not possess the optimal compositional balance required for high pesticide adsorption efficiency. Consequently, the multifactorial optimization of synthesis parameters led to significant improvements, with certain optimized samples exhibiting adsorption performances up to twofold higher than the REF sample. When these results are compared with BET data, an important observation emerges. Although the highest specific surface area was recorded for Sample 1–REF, the most effective materials for practical pesticide adsorption were Sample 7, Sample 8, and Sample 11, which exhibited only moderate specific surface areas. This apparent discrepancy suggests that adsorption efficiency is not governed solely by surface area, but rather by the nature and accessibility of active sites. The enhanced performance of these optimized aerogels is most likely attributable to the optimal presence of Mg^2+^ ions and the homogeneous distribution of Fe_3_O_4_–SA nanoparticles, which collectively increase the density of active adsorption sites, promote larger pore diameters, facilitate access to the mesoporous network, and stabilize interactions with aromatic pesticide molecules [78,79]. Additionally, aerogels with highly aerated, fragile structures, as observed in SEM micrographs for Sample 1–REF, may exhibit reduced structural integrity in aqueous environments, limiting their effective adsorption performance under realistic conditions.

Moreover, the applicability of the optimized aerogel formulations was further demonstrated by systematically evaluating their antimicrobial activity. The use of bacterial isolates originating from contrasting environmental and clinical settings, Danube surface water, raw influent from a municipal wastewater treatment plant, and untreated hospital effluents, provides a realistic framework for assessing antimicrobial performance under complex, variable, and high-load contamination scenarios. In parallel, the inclusion of ATCC reference strains enables direct comparison with internationally standardized susceptibility benchmarks, strengthening the robustness and reproducibility of the conclusions.

The antimicrobial assays revealed clear differences in activity, depending on both the aerogel formulation and the test method. Based on the MIC evaluation against *E. faecalis* and *E. coli*, the most consistent antimicrobial effects were observed for Sample 2, Sample 5, Sample 8, and Sample 11. In contrast, results for *P. aeruginosa* indicated marked resistance to the tested aerogel formulations, with no measurable inhibition detected within the investigated concentration range. A similar trend was observed in the inhibition zone diameter assay. The largest inhibition zones were observed primarily against *E. faecalis* and, to a lesser extent, *E. coli*, with Samples 2, 5, 8, 10, and 11 showing the most pronounced inhibitory effects. Conversely, none of the formulations exhibited relevant antimicrobial activity against *P. aeruginosa*, further confirming the intrinsic resistance profile of this species.

As expected, due to the fundamental differences between planktonic growth inhibition and biofilm-associated behavior, the biofilm inhibition assays yielded distinct patterns. The most consistent antibiofilm activity was observed against *E. faecalis*. Across all three tested concentrations (500, 1000, and 2000 µg/mL), effective biofilm inhibition was recorded particularly for Sample 1, Sample 2, Sample 3, Sample 4, Sample 7, Sample 9, Sample 10, and Sample 11, indicating a robust and concentration-independent antibiofilm response for this species. In the case of *E. coli*, strain-dependent differences became evident when comparing environmental isolates with the reference strain. For the environmental isolates, the lowest tested concentration (500 µg/mL) was generally insufficient to inhibit biofilm formation. However, at 1000 µg/mL, Samples 5, 7, and 11 demonstrated effective antibiofilm activity, while at 2000 µg/mL, all aerogel formulations exhibited measurable biofilm inhibition. In contrast, the ATCC reference strain showed greater sensitivity at lower concentrations, with most aerogels demonstrating antibiofilm activity at 500 µg/mL, except for Samples 6 and 8. At 1000 µg/mL, all formulations were effective against the reference strain, except for Samples 2, 3, and 8. A comparable distinction between environmental and reference strains was also observed for *P. aeruginosa*, although with an inverse trend relative to *E. coli*. For the environmental isolate, antibiofilm activity was detected at 500 µg/mL, primarily in Samples 5 and 10, while at 2000 µg/mL, all aerogels except Sample 8 exhibited biofilm inhibition. In contrast, the reference *P. aeruginosa* strain showed measurable antibiofilm effects only at the highest tested concentration (2000 µg/mL), underscoring the high intrinsic resistance of this species and the concentration-dependent nature of the observed response.

The antimicrobial evaluation results for the aerogels developed and optimized in this study are in good agreement with the existing literature, which consistently shows that nanostructured antimicrobial materials tend to exhibit higher efficacy against Gram-positive bacteria. This behavior is primarily due to the structural characteristics of Gram-positive cell envelopes, which lack an outer membrane barrier and therefore facilitate easier interaction and penetration by antimicrobial agents. In contrast, Gram-negative bacteria possess an additional outer membrane that acts as an effective permeability barrier, significantly restricting intracellular access of many antimicrobial compounds and resulting in reduced or more variable antimicrobial responses [80,81,82,83]. Furthermore, *P. aeruginosa* is widely recognized for its intrinsically high resistance profile, driven by a combination of low outer-membrane permeability, efficient multidrug efflux systems, and pronounced adaptability associated with biofilm formation [52,53,54]. Notably, the most obvious antimicrobial effects were consistently observed for Samples 2 and 5, which were synthesized at the maximum and 75% concentrations of the antimicrobial-active components (Fe_3_O_4_–SA nanoparticles and Mg^2+^ ions), respectively. In addition, Samples 10 and 11 exhibited antimicrobial efficacy comparable to that of Sample 2, and the antimicrobial agent concentrations were similar to those employed in Sample 2. These findings further support the role of both Fe_3_O_4_–SA nanoparticles and Mg^2+^ ions in enhancing the antimicrobial performance of the aerogels, as previously reported for magnetite-based nanomaterials and magnesium-containing antimicrobial systems [84,85,86,87].

Despite systematic variation in reactant mass concentrations during the optimization process, the biological evaluation demonstrated an overall favorable safety profile for all magnesium magnetic silica aerogel formulations. None of the tested samples induced severe cytotoxic effects, inflammatory responses, or pronounced oxidative stress in either HaCaT or HEK293 cells. Importantly, the observed variations in cellular responses remained within biologically acceptable limits and did not exceed thresholds commonly associated with adverse cellular effects. This behavior can be attributed to the intrinsic properties of the components that constitute the magnesium magnetic silica aerogels, each of which contributes to cellular biocompatibility. Silica-based aerogels are widely recognized for their low cytotoxicity and good cellular tolerance, particularly due to their highly porous structure and chemical inertness [88,89].ALG, a biopolymer widely used in biomedical applications such as tissue regeneration and drug delivery, is likewise well known for its excellent biocompatibility and lack of toxicity to mammalian cells [90,91]. Fe_3_O_4_ nanoparticles have also been intensively investigated in medical and biomedical contexts, owing to their multifunctional properties, including favorable cytocompatibility [92,93]. SA is a compound already widely used in human medicine, and when incorporated at suitable concentrations within hybrid materials, it exhibits good cytocompatibility [94,95]. Finally, magnesium ions are essential physiological elements and are broadly recognized for their compatibility with mammalian cells, further supporting the overall biocompatible profile of the developed aerogel systems [96,97]. Although antibacterial activity was evaluated at concentrations up to 2000 µg/mL, cytocompatibility was intentionally assessed at lower concentrations (5–25 µg/mL) that reflect realistic and conservative mammalian exposure scenarios. The developed aerogels are intended for water decontamination as removable solid-phase materials, where high local concentrations are required to inhibit microbial growth, but the material is subsequently separated from the treated water by magnetic recovery. Consequently, direct exposure of human cells to antibacterial working concentrations is not expected, and cytocompatibility testing was focused on biologically relevant exposure levels.

## 4. Materials and Methods

**Figure 23:**
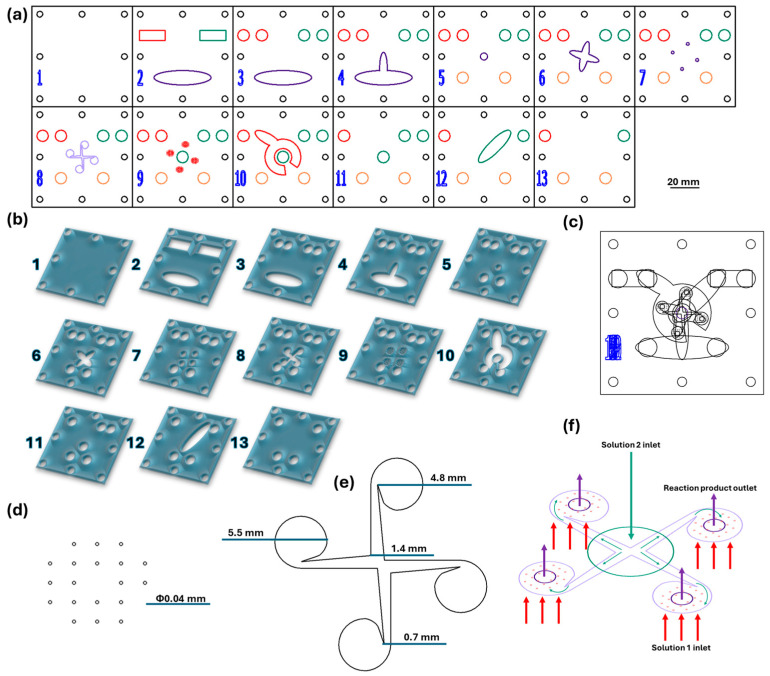
(**a**) Two-dimensional proportional schematic of the individual layers composing the microfluidic platform; scale bar: 20 mm. (**b**) Three-dimensional schematic illustration of the stacked microfluidic layers. (**c**) Superimposed 2D schematic view of the complete microfluidic architecture. (**d**) Detailed dimensions of the reactant inlet channel (layer 9). (**e**) Geometrical dimensions of the vortex-based mixing chamber (layer 8). (**f**) Superimposed view of the reaction zone, where red indicates channels for reagent solution 1 (layer 9), green corresponds to the channel for reagent solution 2 (layer 9), purple denotes the vortex mixing chamber (layer 8), and violet represents the collecting channels (layer 7). Color legend: red—iron precursor inlet channels; green—alkaline medium inlet channels; purple—vortex micromixer; violet—product collection channels and chamber; orange—outlet microchannels. Reprinted from the open-access source [29].

The microfluidic platform (see Figure 23) was fabricated from polymethyl methacrylate (PMMA), purchased as 2 mm-thick plates with a protective film on both sides. The vortex-type chip design was created in RDWorks V8 and sent to a 1610 Pro laser cutting machine (RUBIQ CNC, Bacău, Romania), which precisely cut the components of each layer according to the digital design. Subsequently, the plates were assembled layer by layer to obtain the three-dimensional configuration of the synthesis chip, which was mechanically fixed with screws and sealed using a commercial epoxy adhesive (“Epoxy Universal”, Bison International B.V., Rotterdam, The Netherlands). In the final step, inlet and outlet tubes were inserted to allow for solution flow through the system. The detailed geometrical parameters, operational principles, and microfluidic characteristics of the chip are fully described in our previous publication [29].

For the synthesis of the magnesium magnetic aerogel materials, the following chemical reagents were used: ferric chloride (FeCl_3_) and ferrous sulfate heptahydrate (FeSO_4_·7H_2_O), purchased from Sigma-Aldrich Merck (Darmstadt, Germany); sodium hydroxide (NaOH) from Lach-Ner (Tovarní, Czech Republic); salicylic acid (C_7_H_6_O_3_) from Atochim Prod (Bucharest, Romania); sodium trisilicate (Na_2_O_7_Si_3_), cetyltrimethylammonium bromide (CTAB, C_19_H_42_BrN), sodium alginate (ALG) from brown algae, magnesium chloride (MgCl_2_), ammonium chloride (NH_4_Cl), all obtained from Sigma-Aldrich Merck (Darmstadt, Germany). All solutions were prepared using ultrapure water.

The magnetic Fe_3_O_4_ nanoparticles coated with a salicylic acid shell (Fe_3_O_4_–SA) were synthesized directly within the reaction channels of the microfluidic platform by the simultaneous introduction of Fe^3+^, Fe^2+^, sodium hydroxide, and salicylic acid (SA) solutions. The continuous delivery of the reactive streams was maintained using an osmotic pump (PSP 220 Pump, Model CAR6003, Water Quality Association, Lisle, IL, USA). The Fe_3_O_4_–SA stock dispersion was prepared following the procedure described in our previous study [29], in which the microfluidic parameters and the vortex-induced nanoparticle formation mechanism are fully detailed.

For aerogel synthesis, the same microfluidic chip model was employed, with two reactive solutions simultaneously introduced into the system, which reacted in situ to form the aerogel structure. The first solution was prepared in two stages: (i) in the first stage, sodium trisilicate was mixed with sodium hydroxide in an aqueous medium to obtain the silicate sol; (ii) in the second stage, CTAB was mixed with alginic acid and the Fe_3_O_4_–SA nanoparticle dispersion, yielding a homogeneous suspension. The second solution consisted of magnesium chloride and ammonium hydroxide dissolved and mixed in ultrapure water, serving as the crosslinking and co-precipitation phase.

The synthesis plan was designed according to the methodology established in our previous studies [29,31]. However, in the present work, the protocol was expanded by introducing magnesium chloride as an additional reactant to supply Mg^2+^ ions, enabling further functional enhancement of the magnetic aerogels. Moreover, the synthesis procedure was modified to enable composition optimization by varying the precursor mass ratios, as summarized in Table 6. Five experimental variables were selected due to their significant influence on the properties of the synthesized material: the concentration of magnetic nanoparticle dispersion (Fe_3_O_4_–SA), the concentration of magnesium ions (Mg^2+^), the concentration of ammonium chloride solution (NH_4_Cl), the concentration of alginate solution (ALG), and the concentration of CTAB solution (CTAB). Thus, the initial synthesis procedure, performed in accordance with our previous studies but expanded by incorporating Mg^2+^ ions, was designated as the reference sample for subsequent optimization steps and is referred to as REF. Samples 1–5 were subsequently synthesized by adjusting the concentrations of the reactant solutions according to the optimization design.

**Table 6:** Selection of concentrations for the Fe_3_O_4_–SA nanoparticle solution, magnesium ions (Mg^2+^), ammonium chloride (NH_4_Cl), alginate (ALG), and CTAB solutions. For each component involved in the synthesis, five concentration levels were established, corresponding to minimum, maximum, 50%, 25%, 75%, and reference values. These were determined based on the operational constraints of the microfluidic system and the reaction stability under flow conditions. The selected mass concentration ranges (mg/mL) for the optimization process were as follows: Fe_3_O_4_–SA NPs: 10–70 mg/mL, Mg^2+^: 500–3000 mg/mL, NH_4_Cl: 100–1500 mg/mL, ALG: 50–300 mg/mL, CTAB: 0–1000 mg/mL.

Samples	Mass Concentration of Fe_3_O_4_-SA Nps—Mg^2+^—NH_4_Cl—ALG—CTAB	Observations
Sample 1	10—500—100—50—0	minimum
Sample 2	70—3000—1500—300—100	maximum
Sample 3	40—1750—800—175—50	50.00%
Sample 4	20—875—400—87—25	25.00%
Sample 5	60—2625—1200—262—75	75.00%
REF	33—1690—500—600—80	reference

The next step in the synthesis process involved gelation, achieved by maintaining the suspensions at room temperature for 24 h. Once the samples transitioned to the gel phase, they were washed several times with ultrapure water and ethanol to remove residual ions and unreacted components. The conversion of the gels into aerogels was performed by freeze-drying for 72 h, resulting in completely dried aerogels. The obtained aerogels were physico-chemically characterized using X-ray diffraction (XRD), Fourier-transform infrared spectroscopy (FT-IR), scanning electron microscopy (SEM), energy-dispersive X-ray spectroscopy (EDX), Brunauer–Emmett–Teller (BET), and Fourier-transform ion cyclotron resonance mass spectrometry (FT-ICR HR-MS).

The FT-ICR HR-MS analysis results were used to determine the pesticide adsorption capacity of the synthesized materials. The data were processed using a custom-developed numerical program implementing a hybrid simplex–gradient algorithm to support numerically guided experimental optimization, designed to improve convergence and minimize the number of experimental runs required during the optimization phase. The program incorporated the mass concentrations of the precursors and the experimental results obtained for the six initial samples (Sample 1—REF), in order to identify the optimal synthesis parameters. The objective function used to quantify residual pesticide concentration was defined as follows:

$$f\left(x\right)=\frac{1}{n}\sum_{i=1}^{n}\left(\frac{C_{residual,}\;i(x)}{C_{initial,}\;i}\right)$$

where

*C_initial_* is the initial pesticide concentration in the sample (µg/mL),

*C_residual_* is the residual pesticide concentration after extraction (µg/mL),

*n* represents the number of pesticides analyzed (in this study, *n* = 14).

The corresponding concentrations were obtained from FT-ICR HR-MS analysis of both the fortified pesticide mixture (10–50 ppb) and the extracted samples using the six synthesized aerogels (Sample 1—REF). As a result of this numerically guided experimental optimization approach, five optimized concentration sets were determined for Fe_3_O_4_–SA, Mg^2+^, NH_4_Cl, ALG, and CTAB solutions, leading to the fabrication of aerogels with enhanced pesticide adsorption performance (Samples 7–11). More specifically, this study adopts a performance-driven optimization strategy based on response-guided experimental feedback, with results from FT-ICR HR-MS analyses serving as the primary decision-making tool. Pesticide extraction efficiency was the primary optimization criterion, while antimicrobial properties were evaluated as a complementary functional outcome that further supports the overall performance of the optimized aerogel formulations.

The specific synthesis parameters for the five optimized samples are summarized in Table 7.

**Table 7:** Mass concentrations (mg/mL) of the Fe_3_O_4_–SA nanoparticle solution, magnesium ions (Mg^2+^), ammonium chloride (NH_4_Cl), alginate (ALG), and CTAB solutions, determined after running the objective function within the numerically guided experimental optimization program for aerogel synthesis.

Samples	Mass Concentration of Fe_3_O_4_-Sa Nps—Mg^2+^—NH_4_Cl—ALG—CTAB	Observations
Sample 7	47—1810—1100—50—20	Trial 1
Sample 8	61—2524—1340—200—68	Trial 2
Sample 9	24—1090—461—93—19	Trial 3
Sample 10	70—2800—1400—250—80	Trial 4
Sample 11	50—2300—1200—160—50	Trial 5

The optimization of the formulations was performed in a five-dimensional parametric space defined by the synthesis variables NP, Mg^2+^, NH_4_Cl, alginate, and CTAB. These variables were selected based on their major influence on material formation, stability, and functional performance. For each variable, minimum and maximum admissible values were established according to synthesis feasibility, system stability, and compatibility with purification, characterization, and functional testing procedures. The optimization problem thus involved five degrees of freedom (*n* = 5), with all candidate formulations constrained within the predefined technological domain.

The optimization objective was defined as a scalar experimental performance indicator, derived exclusively from the laboratory synthesis, characterization, and functional evaluation of the materials. This indicator was used consistently throughout the optimization procedure.

The objective function was single-objective, and no multi-objective weighting, aggregation, or computational scoring of individual properties was applied.

In the first stage, the optimization was carried out exclusively through laboratory experiments using a simplex-type strategy based on the Nelder–Mead algorithm. An initial simplex consisting of six experimental points (*n* + 1) was defined within the constrained parameter space. For each simplex point, the corresponding formulation was synthesized, purified, characterized, and functionally tested, and the experimental value of the objective function was determined.

Based on these experimental results, successive simplex transformations were applied, consisting of the reflection of the least-performing point with respect to the centroid of the remaining points. At each iteration, the experimentally measured objective function values were used directly to rank simplex vertices and guide the next experimental step. Four consecutive simplex iterations were performed, each corresponding to a complete experimental cycle. Convergence analysis indicated that further continuation of a purely experimental simplex optimization would require a disproportionate number of additional experiments, incompatible with the available experimental time and resources.

To continue the optimization while reducing experimental effort, a second stage was implemented based on the numerical optimization of an empirical surrogate objective function. All experimental data obtained during Stage I were used to construct a numerical approximation of the experimental response surface. The surrogate objective function was formulated as a low-order multivariate polynomial function of the five synthesis variables, including linear and interaction terms. The surrogate was constructed solely to interpolate the experimentally observed response within the explored parameter domain and was not intended to provide a global prediction. This functional form was selected to ensure numerical stability, smoothness, and consistency with the limited number of available experimental data points.

The surrogate function was defined and used strictly within the experimentally explored parameter domain and did not involve extrapolation beyond this domain. The surrogate objective function was numerically optimized using deterministic constrained optimization procedures, with the same variable bounds as in the experimental optimization. The resulting optimum was treated as a locally optimal estimate and used directly to select the next set of synthesis parameters.

The proposed formulation was subsequently synthesized and experimentally evaluated. The newly obtained experimental result was incorporated into the dataset, and the surrogate function was updated accordingly. The agreement between the surrogate-guided optimum and the experimentally measured objective function value was used as the sole validation criterion for each iteration. This iterative procedure was repeated until a total of eleven experimental points, including the initial simplex, were obtained. The stopping criteria were defined by the convergence of the experimentally measured objective function values and the practical limitations of experimental time and resources.

All formulations obtained during the surrogate-guided stage were therefore experimentally realized and validated, and no formulation was accepted without direct laboratory confirmation.

These optimized parameters were subsequently used to evaluate the structural and functional properties of the synthesized magnesium magnetic aerogels, as discussed in the following section.

### 4.1. Investigation Methods

X-ray diffraction (XRD) was one of the primary techniques employed to investigate the obtained aerogel samples. Measurements were performed using a PANalytical Empyrean diffractometer (PANalytical, Almelo, The Netherlands), equipped with a hybrid monochromator on the incident-beam side and a parallel-plate collimator coupled to a PIXcel 3D detector on the diffracted-beam side. The radiation source was Cu Kα (λ = 1.5406 Å), operated at 40 mA and 45 kV. The analyses were conducted at room temperature using the Grazing Incidence X-ray Diffraction (GIXRD) mode, with an incident angle (ω) of 0.5° and a 2θ scan range between 10° and 80°.

Fourier-transform infrared (FTIR) spectroscopy was employed as a complementary technique for the characterization of the synthesized aerogels. Measurements were performed in ATR-FTIR mode, with the sample mounted on a ZnSe crystal using a Nicolet 6700 FTIR spectrometer (Thermo Fisher Scientific, Waltham, MA, USA). Spectra were recorded in absorbance mode over the 400–4000 cm^−1^ wavenumber range, using 64 scans at a spectral resolution of 4 cm^−1^.

Scanning electron microscopy (SEM) was employed to investigate the morphology and physical dimensions of the synthesized aerogels. The analyses were performed using a Quanta Inspect F50 microscope (Thermo Fisher—FEI, Eindhoven, The Netherlands), equipped with an energy-dispersive X-ray spectroscopy (EDS) module for elemental identification of the samples. Micrographs were acquired by recording the secondary electron signal at an accelerating voltage of 30 keV.

Dynamic light scattering (DLS) analysis was employed to determine the hydrodynamic diameter and zeta potential of the aerogel samples. The materials were dispersed in ultrapure water and subjected to ultrasonic treatment for 5 min to ensure adequate dispersion. Each sample was then injected into the analysis cell of a DelsaMax Pro instrument (Beckman Coulter, Brea, CA, USA), equipped with a 532 nm laser. All measurements were performed in triplicate, and the final results are reported as mean values ± standard deviation. The Brunauer–Emmett–Teller (BET) analysis was employed to determine the specific surface area of the synthesized magnetic aerogels. Measurements were performed using a NOVA 800 gas sorption analyzer (Anton Paar QuantaTec, Inc., Boyton Beach, FL, USA). Prior to analysis, the samples were degassed under vacuum at 180 °C for 4 h. Nitrogen adsorption–desorption isotherms were then recorded at 77 K over a relative pressure range of p/p_0_ = 0.005–1.0. The specific surface area was calculated using the standard BET equation, while the total pore volume was obtained from the adsorbed gas volume at a relative pressure of approximately p/p_0_ ≈ 1. The Barrett–Joyner–Halenda (BJH) model was applied to the desorption branch of the isotherm to estimate the pore-size distribution and mesopore volume.

The role of magnesium magnetic aerogel materials is to treat polluted water, as they exhibit a high adsorption capacity for pesticides and other organic contaminants. For this purpose, six initial samples (Sample 1—REF) with different mass concentrations within predefined experimental ranges were tested, followed by five optimized samples (Sample 7–Sample 11) designed based on the results of the first set. The pesticide adsorption capacity of all samples was assessed in ultrapure water artificially spiked with a pesticide mixture at ppb concentrations. Each magnetic aerogel sample was individually added to the contaminated water and left to interact for 30 min to allow for adsorption to occur. To evaluate adsorption efficiency, the water samples were analyzed before and after treatment by comparing the initial pollutant concentration with the residual concentration after exposure to the aerogel. The investigations were carried out using an FT-ICR HR-MS spectrometer equipped with a 15 T superconducting magnet (SolariX-XR, Bruker Daltonics, Bremen, Germany). Both untreated and treated water samples were introduced via direct infusion under negative electrospray ionization (ESI) mode at a flow rate of 310 μL/h. The nebulizing gas pressure (N_2_) was maintained at 1.5 L/min, with a dry gas flow rate of 2 L/min and a temperature of 210 °C. The spectra were recorded over a mass range of 92–1500 amu, using a source voltage of 4300 V.

As mentioned in the introduction, the literature highlights bacterial contamination of water sources, which poses a major threat to public health and aquatic ecosystems. In this regard, all magnesium magnetic aerogel samples were tested to evaluate their ability to inhibit or reduce the growth of predominant bacterial species commonly found in various water environments. To obtain results that closely reflect real-world conditions, the 11 synthesized samples were tested against both reference bacterial strains from the ATCC (American Type Culture Collection), representing the international standard, and bacteria isolated from different water sources. For this study, bacterial strains from three relevant species were used: *Enterococcus faecalis* (Gram-positive), *Escherichia coli*, and *Pseudomonas aeruginosa* (both Gram-negative). The strains were selected from the Microbiology Laboratory collection of the Faculty of Biology, University of Bucharest. The set included both recent isolates from aquatic environments and internationally recognized reference strains. *E. faecalis* strains E7 and E10, along with *E. coli* strains CCE1D, DCE2D, and CE1D, were isolated in July 2024 from surface water samples collected downstream of the Glina Wastewater Treatment Plant. The *P. aeruginosa* strain Ps 25 originated from raw urban wastewater collected from the influent of the Glina WWTP, while strain Ps 36 was isolated from non-chlorinated hospital wastewater obtained from the Sf. Paraschiva Infectious Diseases Hospital in Iași. In parallel, the ATCC reference strains *E. faecalis* ATCC 29212, *E. coli* ATCC 25922, and *P. aeruginosa* ATCC 27853 were also used. The details regarding the bacterial species, their origin, and experimental testing conditions are summarized in Table 8.

### 4.2. Determination of Minimum Inhibitory Concentration (MIC)

For the quantitative evaluation of antimicrobial activity, the binary serial microdilution method was employed in sterile 96-well microtiter plates. The same bacterial selection was used as in the qualitative assays, environmental isolates (Glina, Danube, hospital) and ATCC reference strains, covering all three investigated bacterial species. Each tested compound (aerogel or bioactive nanosystem) was prepared at an initial concentration of 2 mg/mL and added to the first well of each row. Subsequently, eight two-fold serial dilutions were performed using a micropipette, starting from well 1A and ending in well 8H, where the final concentration reached 0.015625 mg/mL. A volume of 15 µL of bacterial suspension standardized to 0.5 McFarland was added to each well. The plates were incubated for 24 h at 37 °C. After incubation, the MIC value was determined visually by identifying the lowest concentration at which no turbidity or visible bacterial growth was observed. The same experimental setup included control wells to ensure result accuracy: bacteria + DMSO (solvent control), medium without bacteria (negative control), and bacteria without treatment (strain control).

### 4.3. Qualitative Analysis of Antimicrobial Effect

Bacterial strains were preserved in nutrient broth supplemented with 20% glycerol and stored at –80 °C. Prior to testing, the strains were subcultured on nutrient agar plates. The resulting colonies were suspended in sterile physiological saline (SPS) until a turbidity equivalent to 0.5 McFarland standard was reached, corresponding to a bacterial density of approximately 1–3 × 10^8^ CFU/mL. The assay was based on a modified Kirby–Bauer disk diffusion method. A uniform layer of solid nutrient medium was poured into Petri dishes, and the bacterial suspension was evenly spread over the surface using a sterile swab. Subsequently, 5 µL drops of each tested formulation (aerogel diluted to 20 mg/mL in sterile DMSO) were placed at equal distances of approximately 3 cm. The Petri dishes were incubated for 18–24 h at 37 °C, after which the diameters of the bacterial growth inhibition zones surrounding each drop were measured.

### 4.4. Assessment of Monospecific Biofilm Formation

The impact of the tested formulations on bacterial biofilm development was assessed using a liquid-phase serial microdilution protocol, similar to that employed for MIC determination. Following serial dilutions (ranging from 2 mg/mL to 0.015625 mg/mL), 15 µL of bacterial suspension standardized to 0.5 McFarland was added to each well. The microtiter plates were incubated for 24 h at 37 °C to allow for biofilm formation. After incubation, the plates were washed three times with sterile physiological saline to remove non-adherent cells and fixed with cold methanol for 5 min. The formed biofilms were then stained with 1% crystal violet solution for 20 min. Excess dye was removed with tap water, and the biofilm-bound dye was subsequently solubilized with 33% acetic acid solution. The resulting solutions were analyzed spectrophotometrically at 490 nm, and the absorbance values were used to quantify the biofilm density.

### 4.5. Cell Culture

The HEK293 human embryonic kidney cell line was obtained from the American Type Culture Collection (ATCC, cat. no. CRL-1573). The HaCaT human keratinocyte cell line was purchased from Cell Lines Service (CLS, Germany, cat. no. 300493). Both cell lines were cultured according to the suppliers’ recommendations and routinely monitored for morphology and contamination. The biological response of cells in contact with the tested materials was assessed through in vitro biocompatibility evaluation using two human cell lines: HEK293 (human embryonic kidney cells) and HaCaT (human keratinocytes). The cells were incubated for 48 h with the magnetic aerogel suspensions prepared in the appropriate culture medium for each cell line. For test validation, control wells containing cells not exposed to any material suspension were included and served as the negative control.

### 4.6. MTT Cell Viability Assay

After 48 h of incubation of the cell cultures with various aerogel concentrations, the culture medium was removed from each well, and the cells were washed with 100 µL of PBS to eliminate residual material. Subsequently, 130 µL of MTT solution at a concentration of 1 mg/mL was added to each well. Following 90 min of incubation at 37 °C and 5% CO_2_, the MTT solution was aspirated, and the resulting formazan crystals in each well were solubilized with 130 µL of 100% isopropanol. Absorbance was measured at 595 nm using a FlexStation 3 microplate reader. The color intensity is directly proportional to the number of metabolically active cells, indicating cell viability.

### 4.7. Determination of Nitric Oxide (NO) Levels

After 48 h of incubation of the cells with different aerogel concentrations, 80 µL of supernatant from each well was transferred into a new 96-well microplate. The Griess colorimetric reaction was used to quantify nitric oxide (NO) production. A freshly prepared reagent mixture containing 1% sulfanilamide solution (S) and 0.1% N-(1-naphthyl)ethylenediamine dihydrochloride solution (N) was added in a 1:1 (*v*/*v*) ratio to each sample (80 µL per well). Absorbance was measured at 550 nm using a FlexStation 3 microplate reader, and the obtained values reflected the relative amount of nitric oxide (NO) released by the cells.

### 4.8. Determination of Lactate Dehydrogenase (LDH) Release

After 48 h of incubation of the cells with different aerogel concentrations, 50 µL of supernatant from each well was transferred into a new 96-well microplate. To each sample, 50 µL of the reaction mixture (prepared by mixing equal volumes of catalyst solution and dye solution) was added. The plate was then incubated for 30 min at room temperature in the dark to allow the enzymatic reaction to proceed. Absorbance was measured at 490 nm using a FlexStation 3 microplate reader. The signal intensity was proportional to the amount of LDH released into the medium, indicating the degree of cell membrane damage and thus the cytotoxic potential of the tested materials.

### 4.9. Determination of Intracellular Reactive Oxygen Species (ROS) Levels

At the end of the exposure period, the culture medium was removed from the plates, and the cells were incubated with 10 µM 2′,7′-dichlorodihydrofluorescein diacetate (H_2_DCFDA) prepared in culture medium, for 30 min at 37 °C. After incubation, the H_2_DCFDA-containing medium was discarded, and the cells were washed with PBS to remove any unbound or non-internalized dye. Fluorescence intensity was measured using a fluorimeter at an excitation wavelength of 485 nm and an emission wavelength of 515 nm. The obtained values were normalized to viable cell fluorescence and expressed relative to untreated control samples. Additionally, background fluorescence resulting from nonspecific binding of the fluorochrome to the plate surface (in the absence of cells) was subtracted from the values obtained in cell-containing wells to ensure data accuracy.

### 4.10. LIVE/DEAD™ Cell Viability Assay

To evaluate cell viability and cytotoxicity, calcein-AM and ethidium homodimer-1 solutions from the LIVE/DEAD™ Viability/Cytotoxicity Kit (ThermoFisher, Waltham, MA, USA) were used according to the manufacturer’s instructions. After 30 min of incubation with the dye mixture, the cells were visualized using an Olympus IX73 fluorescence microscope (Olympus, Tokyo, Japan). Viable cells exhibited a bright green fluorescence due to calcein-AM conversion, while non-viable cells showed red fluorescence from ethidium homodimer-1 binding to nuclear DNA, indicating loss of membrane integrity.

### 4.11. Analysis of Cellular Morphological Changes by Phalloidin-FITC Staining

In this study, cytoskeletal morphological alterations induced by aerogel exposure were visualized using phalloidin conjugated with FITC (fluorescein isothiocyanate). After removal of the culture medium, the cells were fixed in the wells with 100 µL/well of 4% paraformaldehyde in PBS for 20 min at room temperature. Subsequently, cell membranes were permeabilized with 100 µL/well of 0.1% Triton X-100—1.2% BSA in PBS for 1 h. After three PBS washes, the cells were incubated for 1 h with 130 µL of phalloidin–FITC solution (20 µg/mL) to stain actin filaments. The cell nuclei were then counterstained with 150 µL/well of DAPI solution (2 µg/mL) for 15 min, followed by three PBS washes. Cells were visualized using an Olympus IX71 inverted fluorescence microscope, and the resulting images were analyzed to assess morphological changes and actin filament organization following nanoparticle exposure.

### 4.12. Preparation of Cell Lysates

For the preparation of cell lysates, cells treated with different aerogel concentrations were first detached and then centrifuged at 1500 rpm for 10 min at 18 °C to collect the cell pellet. The supernatant was discarded, and the resulting pellet was washed with 4 mL of sterile PBS and centrifuged again under the same conditions. After removing the supernatant, the cell pellet was resuspended in 300 µL of PBS. Cell lysis and extraction of intracellular proteins were achieved by sonication (3 cycles of 30 s each, performed on ice). The samples were subsequently centrifuged at 3000 rpm for 15 min at 4 °C to remove cellular debris. The protein concentration in the cell lysates was determined using the Bradford reagent.

### 4.13. Determination of Malondialdehyde (MDA) Levels

A reliable and widely used method for monitoring lipid peroxidation involves the reaction of thiobarbituric acid (TBA) with malondialdehyde (MDA). The resulting MDA–TBA adducts, formed at 37 °C, were quantified fluorometrically at 520 nm excitation and 549 nm emission. A 1 µM MDA standard solution was used to prepare a calibration curve, with serial dilutions ranging from 0 to 0.5 µM, using 0.1 N HCl as diluent. For each sample (standard or appropriately diluted total protein extract), 200 µL of the sample was mixed with 700 µL of 0.1 N HCl, homogenized, and left to stand at room temperature for 20 min. Subsequently, 900 µL of 0.025 M TBA solution was added to each reaction tube, followed by incubation at 37 °C for 65 min to allow for the formation of TBA–MDA condensation products. For the MDA standards, 400 µL of bovine serum albumin (BSA) solution in PBS (concentration adjusted according to protein content) was added, while 400 µL of PBS was used for the cell lysate samples. Fluorescence intensity was measured using a FlexStation 3 microplate reader, and an MDA calibration curve was generated by plotting MDA concentration versus fluorescence (RFU). The fluorescence values of the cell lysate samples were extrapolated from the standard curve to yield their MDA concentrations. The degree of lipid peroxidation for each sample was normalized to total protein concentration, as determined by the Bradford assay.

### 4.14. Determination of Reduced Glutathione (GSH) Levels

The concentration of reduced glutathione (GSH) was determined by a spectrophotometric assay based on the reaction of 5,5′-dithiobis(2-nitrobenzoic acid) (DTNB) with GSH. In this reaction, DTNB is reduced to 5-thio-2-nitrobenzoic acid (TNB), a yellow-colored product whose absorbance can be measured spectrophotometrically. The amount of TNB formed is directly proportional to the oxidized GSH concentration, and absorbance was recorded at 405 nm. Initially, the cell lysates were deproteinized by mixing with a 5% sulfosalicylic acid (SSA) solution in a 1:1 (*v*/*v*) ratio. The mixture was then centrifuged at 5000 rpm for 10 min at 4 °C, and the supernatant was transferred (10 μL) into a 96-well microplate. To each well, 150 μL of reaction mixture was added, containing 100 mM potassium phosphate buffer (pH 7.0), 1 mM EDTA, and 1.5 mg/mL DTNB solution. The plate was incubated for 10 min at room temperature, and absorbance was measured at 412 nm. The GSH concentration was determined from a calibration curve constructed using known concentrations of GSH standard solutions.

### 4.15. Statistical Analysis

All syntheses were performed under identical microfluidic conditions to ensure reproducibility of the material formulations. For each aerogel composition, a single representative synthesis was performed, followed by multiple independent analytical measurements, depending on the technique employed. Physico-chemical analyses were performed as single measurements per formulation, except for DLS analyses, which were performed in triplicate (*n* = 3, and results are expressed as mean ± standard deviation (SD)). All microbiological relevant and biological experiments were performed in triplicate (*n* = 3), and results for treated samples were normalized to controls. Data analysis and graphical representation were performed using Microsoft Excel, and values are expressed as mean ± standard deviation (SD). Statistical significance (only for biological assay) was assessed using Student’s t-test, with differences considered significant at *p* < 0.05 (* *p* < 0.05, ** *p* < 0.01, *** *p* < 0.001).

## 5. Conclusions

In this study, magnesium magnetic silica aerogels were successfully synthesized using a microfluidic platform that enables precise control over reactant interactions and final material composition. The developed aerogels are based on a mesoporous silica network enhanced with alginate, Mg^2+^ ions, and salicylic-acid-functionalized Fe_3_O_4_ nanoparticles, resulting in hybrid materials with advanced and multifunctional properties. An aerogel optimization strategy combining theoretical design and reactant concentrations selected via numerically guided experimental optimization was employed, yielding 11 distinct samples with different physicochemical characteristics and biological activities. Comprehensive physicochemical characterization using XRD, FTIR, SEM, EDS, and BET analyses confirmed the successful integration of all constituent components into a uniform mesoporous silica framework, while also highlighting the critical role of the optimization process in tailoring material properties. Importantly, the results demonstrated that aerogels exhibiting the highest specific surface areas, typically those synthesized using theoretically defined concentrations, did not necessarily display the best performance in pesticide adsorption, as evaluated by FT-ICR HR-MS. Instead, aerogels obtained using compositions derived from numerically guided experimental optimization showed superior functional performance. Sample 7, Sample 8, and Sample 11 exhibited enhanced pesticide adsorption efficiencies despite having moderate BET surface areas, emphasizing that adsorption performance is governed not only by surface area, but also by pore accessibility, active site density, and the chemical affinity imparted by Mg^2+^ ions and Fe_3_O_4_–SA nanoparticles. Regarding the antimicrobial activity, valuable insights were obtained that further underline the influence of aerogel optimization. The most pronounced antimicrobial effects were observed against *Enterococcus faecalis* (strong activity), followed by *Escherichia coli* (modest activity)*,* while *Pseudomonas aeruginosa* exhibited the highest resistance (no effects), in agreement with its well-documented intrinsic defense mechanisms. Equally important, biological evaluations using HaCaT and HEK293 cell lines demonstrated the aerogels’ good cytocompatibility, supporting their safe interaction with mammalian cells and confirming that the compositional variations introduced during optimization did not induce adverse cellular effects.

Overall, this study demonstrates that microfluidic-assisted synthesis, combined with chemical optimization, represents an effective and versatile strategy for developing multifunctional aerogels capable of addressing both chemical and biological water contamination. Magnesium magnetic silica aerogels are promising candidates for advanced water treatment applications, offering efficient pollutant removal, antimicrobial efficacy, and convenient magnetic recovery.

## Figures and Tables

**Table 1:** Numeric data of hydrodynamic diameter (nm).

Hydrodynamic Diameter (nm)										
Sample 1	Sample 2	Sample 3	Sample 4	Sample 5	REF	Sample 7	Sample 8	Sample 9	Sample 10	Sample 11
2406.9	1469.27	1708.83	594.3	637.53	1836.57	404.7	1300	2029.3	433.53	793.47

**Table 2:** Numeric data of zeta potential (mv).

Zeta Potential (mv)										
Sample 1	Sample 2	Sample 3	Sample 4	Sample 5	REF	Sample 7	Sample 8	Sample 9	Sample 10	Sample 11
−17.90	−21.20	−19.76	−18.32	−19.30	−17.56	−18.04	−20.89	−18.13	−24.44	−21.30

**Table 3:** Effect of selected mass concentrations of Fe_3_O_4_–SA nanoparticles, Mg^2+^, NH_4_Cl, alginate (ALG), and CTAB on the pore characteristics of magnesium magnetic silica aerogels (Samples 1–11), including specific surface area, pore volume, and average pore diameter.

Mass Concentration of Fe_3_O_4_-SA Nps—Mg^2+^—NH_4_Cl—ALG—CTAB	Surface	Pore Volume	Pore Diameter
10—500—100—50—0 (Sample 1—minimum)	615.7 m^2^/g	0.939 cm^3^/g	6.10 nm
70—3000—1500—300—100 (Sample 2—maximum)	357.1 m^2^/g	0.565 cm^3^/g	6.33 nm
40—1750—800—1750—50 (Sample 3—50.00%)	391.8 m^2^/g	0.605 cm^3^/g	6.18 nm
20—875—400—87—25 (Sample 4—25.00%)	436.3 m^2^/g	0.773 cm^3^/g	7.09 nm
60—2625—1200—262—75 (Sample 5—75%)	315.2 m^2^/g	0.610 cm^3^/g	7.74 nm
33—1690—500—600—80 (REF—reference)	539.5 m^2^/g	0.845 cm^3^/g	6.26 nm
47—1810—1100—50—20 (Sample 7—Trial 1)	294.3 m^2^/g	0.570 cm^3^/g	7.74 nm
61—2524—1340—200—68 (Sample 8—Trial 2)	243.7 m^2^/g	0.509 cm^3^/g	8.56 nm
24—1090—461—93—19 (Sample 9—Trial 3)	250.0 m^2^/g	0.541 cm^3^/g	8.66 nm
70—2800—1400—250—80 (Sample 10—Trial 4)	127.8 m^2^/g	0.329 cm^3^/g	10.28 nm
50—2300—200—160—50 (Sample 11—Trial 5)	200.2 m^2^/g	0.428 cm^3^/g	8.54 nm

**Table 4:** Mass spectrometric characteristics of the pesticide standards included in the study: molecular formula, detected *m*/*z* values, ion species, and charge state (z).

Pesticide	Molecular Formula	*m*/*z*	Ion Form	z
**Alaclor**	C_14_H_20_C_l_NO_2_	292.1074	M + Na	+1
**Bromopropylate**	C_17_H_16_Br_2_O_3_	410.9412	M − OH	−1
**Cypermethrin**	C_22_H_19_C_l2_NO_3_	416.08151	M + H	+1
**EPN**	C_14_H_14_NO_4_PS	324.0454	M + H	+1
**Fenpropathrin**	C_22_H_23_NO_3_	350.17507	M + H	+1
**Fensulfothion**	C_11_H_17_O_4_PS_2_	309.03786	M + H	+1
**Paraoxon ethyl**	C_10_H_14_NO_6_P	276.06312	M + H	+1
**Phosalone**	C_12_H_15_C_l_NO_4_PS_2_	367.99409	M + H	+1
**Phosmet**	C_11_H_12_NO_4_PS_2_	318.00178	M + H	+1
**Propyzamide**	C_12_H_11_C_l2_NO	256.02901	M + H	+1
**Pyrazophos**	C_14_H_20_N_3_O_5_PS	374.09337	M + H	+1
**Tebufenpyrad**	C_18_H_24_C_l_N_3_O	334.16804	M + H	+1
**Triazophos**	C_12_H_16_N_3_O_3_PS	314.07227	M + H	+1

**Table 5:** Extraction efficiency (%) and average extraction efficiency (%) of each aerogel formulation for the pesticide mixture, calculated based on the initial and residual concentrations measured by FT-ICR HR-MS.

Sample	Alaclor	Bromopropylate	Cypermethrin	EPN	Fenpropathrin	Fensulfothion	Paraoxon ethyl	Phosalone	Phosmet	Propyzamide	Pyrazophos	Tebufenpyrad	Triazophos	Average
Sample 1	85.65633	3.24949	21.88848	11.91119	42.61378	50.09619	51.60827	61.62776	90.62975	55.40124385	57.0179376	58.9927019	40.51549	48.55451
Sample 2	39.238	3.509036	53.97381	23.14772	39.50928	40.88421	70.27508	49.8687	88.97206	69.23511281	26.9998403	47.4699407	31.29224	44.95192
Sample 3	83.27238	22.72746	42.50768	3.246051	34.95757	44.56783	72.74888	59.10998	89.28867	62.37626732	40.5561158	55.3670019	32.04022	49.44355
Sample 4	79.03611	33.4137	31.83793	7.184314	42.85233	55.20468	59.77116	67.86236	92.50157	63.9110011	55.5616025	56.700661	45.86725	53.20805
Sample 5	89.41548	32.76371	47.4676	35.71472	54.06468	64.89085	81.21085	71.23306	93.27972	72.97214313	55.7346806	64.8085315	45.58579	62.24168
REF	77.49132	−9.34519	16.30078	0.605223	25.71539	28.4295	15.04426	49.63704	89.73962	34.01946243	25.3974744	39.452583	7.902739	30.79925
Sample 7	32.62806	80.21247	67.31911	79.7305	78.24469	57.07472	72.7818	67.72685	3.579737	68.16558669	82.6653146	71.9211327	78.74139	64.67626
Sample 8	86.32288	42.12689	65.41663	70.48298	84.46112	69.87213	74.05052	80.18122	90.55522	81.01704574	21.6209239	31.285121	54.41501	65.52367
Sample 9	98.46372	51.66458	53.22449	45.1984	60.12213	64.19216	47.11391	46.38883	80.04226	52.67130917	−34.9317396	31.3288578	44.21983	49.20759
Sample 10	74.38163	35.43757	50.03366	38.46345	40.06697	49.33946	55.89242	65.09001	52.09001	69.84573518	29.169514	48.0500076	43.40864	50.15937
Sample 11	80.37746	52.71428	56.88352	47.77476	53.37695	70.41693	56.76314	72.97095	51.54969	78.1178736	76.6425671	74.3157001	79.56629	65.4977

**Table 8:** Bacterial strains used in the study and their sources of origin. The table includes both isolated strains obtained from various water sources (urban, hospital, and surface wastewater) and internationally recognized reference strains from the ATCC (American Type Culture Collection).

Code	Strains	Bacterial Strain Collection Code	Source of Isolation/ Sampling Points
Ec. 1	*E. coli*	CCE1D	Strain isolated in July 2024 from downstream water sample—Glina
Ec. 2	*E. coli*	DCE2D	Strain isolated in July 2024 from downstream water sample—Glina
Ec. 3	*E. coli*	CE1D	Strain isolated in July 2024 from downstream water sample—Glina
Ec. 4	*E. coli*	ATTC 25922	-
Ps. 1	*P. aeruginosa*	Ps 25 (20082 CNE6) Wastewater (urban)	Glina Wastewater Treatment Plant, Bucharest—(raw/untreated wastewater)
Ps. 2	*P. aeruginosa*	Ps 36 (20096 CK2) Wastewater (hospital)	Hospital wastewater—untreated (non-chlorinated) effluent/collector channel of the Sf. Paraschiva Infectious Diseases Hospital, Iași
Ps. 3	*P. aeruginosa*	ATTC 27853	-
En. 1	*E. faecalis*	E7 (water)	Surface water (Danube River)
En. 2	*E. faecalis*	E10 (water)	Surface water (Danube River)
En. 3	*E. faecalis*	ATTC 29212	-

## Data Availability

The original contributions presented in this study are included in the article. Further inquiries can be directed to the corresponding author.
